# Apocrine Secretion in *Drosophila* Salivary Glands: Subcellular Origin, Dynamics, and Identification of Secretory Proteins

**DOI:** 10.1371/journal.pone.0094383

**Published:** 2014-04-14

**Authors:** Robert Farkaš, Zuzana Ďatková, Lucia Mentelová, Péter Löw, Denisa Beňová-Liszeková, Milan Beňo, Miklós Sass, Pavel Řehulka, Helena Řehulková, Otakar Raška, Lubomír Kováčik, Jana Šmigová, Ivan Raška, Bernard M. Mechler

**Affiliations:** 1 Laboratory of Developmental Genetics, Institute of Experimental Endocrinology, Slovak Academy of Sciences, Bratislava, Slovakia,; 2 Department of Genetics, Comenius University, Bratislava, Slovakia; 3 Department of Anatomy and Cell Biology, Lorand Eötvös University, Budapest, Hungary; 4 Institute of Molecular Pathology, Faculty of Military Health Sciences, University of Defence, Hradec Králové, Czech Republic; 5 1st Department of Internal Medicine - Cardioangiology, Faculty of Medicine in Hradec Králové, Charles University in Prague, Hradec Králové, Czech Republic; 6 Institute of Cellular Biology and Pathology, 1st Faculty of Medicine, Charles University in Prague, Prague, Czech Republic; 7 Deutsches Krebsforschungszentrum, Heidelberg, Germany; Technische Universität Dresden, Germany

## Abstract

In contrast to the well defined mechanism of merocrine exocytosis, the mechanism of apocrine secretion, which was first described over 180 years ago, remains relatively uncharacterized. We identified apocrine secretory activity in the late prepupal salivary glands of *Drosophila melanogaster* just prior to the execution of programmed cell death (PCD). The excellent genetic tools available in *Drosophila* provide an opportunity to dissect for the first time the molecular and mechanistic aspects of this process. A prerequisite for such an analysis is to have pivotal immunohistochemical, ultrastructural, biochemical and proteomic data that fully characterize the process. Here we present data showing that the *Drosophila* salivary glands release all kinds of cellular proteins by an apocrine mechanism including cytoskeletal, cytosolic, mitochondrial, nuclear and nucleolar components. Surprisingly, the apocrine release of these proteins displays a temporal pattern with the sequential release of some proteins (*e.g*. transcription factor BR-C, tumor suppressor p127, cytoskeletal β-tubulin, non-muscle myosin) earlier than others (*e.g*. filamentous actin, nuclear lamin, mitochondrial pyruvate dehydrogenase). Although the apocrine release of proteins takes place just prior to the execution of an apoptotic program, the nuclear DNA is never released. Western blotting indicates that the secreted proteins remain undegraded in the lumen. Following apocrine secretion, the salivary gland cells remain quite vital, as they retain highly active transcriptional and protein synthetic activity.

## Introduction

Secretory release is the process by which cells selectively externalize compounds as a part of numerous metabolic exchanges, and is considered to be a basic feature of every eukaryotic cell. One type of widespread and well known secretory process is exocytosis, whose intensely studied mechanism has identified many dozens of factors [1]–[17]. Exocytosis is the process regulating the specific membrane contact, priming and fusion events required for the selective release of compartmentalized compounds such as signaling molecules (morphogens, growth factors, antibodies, neurotransmitters, cytokines, hormones, *etc*.). The exocytotic secretory pathway involves the formation of vesicles in the *trans*-Golgi in its initial phase, then targeted translocation of these vesicles to sites on the plasma membrane, the preparation of these docked vesicles for full fusion competence (priming), and the subsequent triggered fusion of these membranes, resulting in their coalescence and the release of vesicular contents to the extracellular space. A complex composed of three major membrane proteins, each representing a small protein family conserved from yeast to humans, has emerged as key player in exocytosis [18]–[21]. The hexameric ATPase NSF (*N*-ethylmaleimide-sensitive fusion protein) is capable of putting energy into the system. Members of the SNAP (soluble NSF-attachment protein) family appear to function as adaptors between NSF and the third type of protein in the complex, the SNAREs (SNAP receptors). SNAREs are found on both the target membrane (t-SNAREs) and the vesicle (v-SNAREs) and are therefore assumed to be the major “targeting” components of the process [22]–[24].

In addition to exocytosis, which takes place by targeted fusion of secretory vesicles with the plasma membrane, there exist two additional types of noncanonical secretion: apocrine and holocrine secretion during which entire portions of the cell are released and homotypic membrane fusion is not required. In the apocrine mechanism, a glandular cell loses a portion of its cytoplasm and is then completely or partially renewed. In the case of holocrine secretion, the material is released into the gland lumen upon cell death and the dissolution of cellular structure. In contrast to exocytosis (merocrine secretion), no protein components, factors or genes affecting apocrine and/or holocrine secretion have yet been identified, and thus the mechanisms underlying these processes remain enigmatic.

In textbooks and reviews, apocrine secretion is frequently described either in association with the lactation activity of mammary glands, the Harderian gland, and some exocrine glands [25]–[27] or notably as a differential diagnostic marker for some benign metaplasias and in many dermatogenic and some breast cancers [28]–[37].

Apocrine secretion was first described 180 years ago in 1833 when Purkinje [38] discovered the process in human sweat glands, a typical apocrine secretory organ. Independently, Velpeau [39] and later Verneuil [40] described a chronic acneiform infection of the cutaneous apocrine glands that has been named hidradenitis suppurativa (HS) [41]–[46]. Despite this, until now we have no understanding of the proteins and corresponding genes involved in apocrine or holocrine secretion at the level of their control, origin or contents of the secretagogue. Even the morphological description of the process is traditionally and often incorrectly just transferred from one textbook to another without referencing any primary literature. Consequently, even though the literature on apocrine and holocrine secretion accounts for more than 95, 000 original papers in Medline Pubmed and Web of Science databases, most refer to associated pathologies, and the mechanisms underlying these types of secretion remain outside of the interests of mainstream research.

During a set of experiments on programmed cell death (PCD) in *Drosophila* in our laboratory, we discovered that the doomed larval salivary glands release proteins by an unusual extrusion process during the late prepupal period [47]. We show here that this hitherto neglected protein extrusion process, which takes place just 6 to 4 hr prior to execution of PCD, occurs via a typical apocrine mechanism. Not only is this the first description of apocrine secretion in *Drosophila*, the rich array of methods and molecular-genetic tools available in the fruitfly offers an outstanding opportunity to dissect the mechanism of this process and identify the genes regulating it. As a prerequisite towards this goal, we present here the light and electron microscopical evidence for the apocrine process in the prepupal salivary glands, describe its dynamics, and characterize the secreted proteins.

## Materials and Methods

### Fly culture and genotypes

Flies were cultured in 50 ml vials or 200 ml bottles at 23°C on agar-yeast-cornmeal-molasses medium [48], [49] with the addition of methylparaben to prevent molds. Observations were carried out on 3^rd^ instar larvae and prepupae of *Drosophila melanogaster* (Meigen) wild type strain *Oregon R* originally obtained from Umea Drosophila Stock Centre, Umea, Sweden, was used as standard reference control [50].

Following fluorescent protein-traps or fusion protein insertion lines were used: *RFP-histone 3* (Kami Ahmad, Harvard Medical School, Boston, USA), *RFP-Sgs3* (Andy Andres, University of Nevada, Las Vegas, USA), *GFP-clathrin*, *GFP-Atg5*, *GFP-Atg8* (Tom Neufeld, University of Minnesota, Minneapolis, USA), *GFP-LC3* (Tor-Erik Rusten, The Norwegian Radiumhospital, Oslo), *UAS-tauGFP*, *UAS-GFP-LAMP1* (Helmut Krämer, University of Texas Southwestern Medical Center at Dallas, USA) *hs-GFP-moesin* (Dan Kiehart, Duke University, Durham, NC, USA). Then *GFP-RNP 87F squid*, *GFP-Rbp1*, *GFP-VhaSFD*, *GFP-Pdi*, *GFP-Grasp65*, *GFP-Atpα* (α-subunit of Na^+^,K^+^-ATPase), *GFP-Corail*, *GFP-Luciole* (UDP-glycosyltransferase), *GFP-Spider* (*gilgamesh*; Ser/Thr casein kinase), *GFP-shaggy* (zw3 Ser/Thr kinase), *GFP-Rtc1* (RNA-binding RNA-3'-phosphate cyclase), *GFP-Résille* (Aldo/keto reductase), *GFP-Cocoon* (Chaperonin Cpn60 ATPase), *GFP-MA3-like* (RCC1-like & MA3-like RNA binding protein), *GFP-Coconut* (Hsp20-like α-crystallin), *GFP-Thor* (tropomyosin 1/prefoldin), *GFP- βTub56D*, *GFP-Hrb98DE* and *scribbler* (Alain Debec, CNRS, Villefranche sur mer, France). For complete list of fly stocks used in this study see Tables 1, 2 and 3. All other GFP-insertion lines in this work were from William Chia (Institute of Molecular and Cell Biology, Singapore), Michael Buszczak (University of Texas Southwestern Medical Center at Dallas, USA), and Bloomington Stock Center.

**Table 1:** List of proteins released by apocrine secretion and detected by antibodies using immunostaining.

Protein	Corresponding gene	MW (kDa)	Function/Cellular localization	Detection method	Time of release (hr APF)
Actin	*Act5C* + *Act42A*	41.8	cytoskeletal/cortical, apical	antibody/phalloidin	8 and 9.5
Arm	*armadillo*	93.0	cytoskeletal, signaling/membrane, cytoplasmic	antibody	9
Baz	*bazooka*	157.4	asymmetric division/cortical, apical	antibody	9–10
BR-C	*Broad-Complex*	77.4	transcription and chromatin remodeling factor/nucleus	antibody	9
α-Catenin	*α-Catenin*	110.0	cytoskeletal/membrane and cytoplasmic	antibody	8
Crb	*crumbs*	234.0	cytoskeletal/apical	antibody	9
DHR78	*Drosophila hormone receptor in 78*	65.4	nuclear receptor, transcription factor/nucleus	antibody	9
Dlg	*discs large*	102.0	tumor suppressor/membrane	antibody	8–9
Doa	*Darkener of apricot*	55.0	dual-specific protein kinase/cytoplasmic and nuclear	antibody	8
E-cadherin	*shotgun*	150.0	cytoskeletal and signaling/membrane	antibody	9
EcR	*Ecdysone receptor*	94.0	nuclear receptor, transcription factor/nucleus	antibody	8
E63	*Ecdysone-induced protein 63F/E63-1*	22.0	calcium binding EF hand/cytoplasmic, secretory	antibody	8
E74	*Ecdysone-induced protein 74EF/E74*	87.1	transcription factor/nucleus	antibody	9
E75	*Ecdysone-induced protein 75B/E75*	147.2	nuclear receptor, transcription factor/nucleus	antibody	8
Fasciclin I	*Fasciclin I*	72.6	cell adhesion, signaling/cell membrane	antibody	9
Fasciclin III	*Fasciclin III*	55.8	cell adhesion, signaling/cell membrane	antibody	9
Fibrillarin	*Fibrillarin*	34.6	RNA processing/nucleolus	antibody	9
βFTZ-F1	*ftz transcription factor1*	95.0	nuclear receptor, transcription factor/nucleus	antibody	9
Imp-α1	*Importin α1*	60.0	protein transport/cytoplasmic, nucleus	antibody	8
Kr-H	*Kruppel homolog 1*	91.5	transcription factor/nucleus	antibody	9
Malic enzyme	*Malic enzyme*	84.0	malate dehydrogenase/cytoplasmic	antibody	
Met	*Methoprene-tolerant*	79.0	transcription factor/nucleus	antibody	9
Mitochondrial pyruvate dehydrogenase	*l(1)G0334*	43.9	pyruvate dehydrogenase/mitochondria	antibody	8
Non-muscle myosin II heavy chain	*zipper*	227.0	cytoskeletal	antibody	8
Nuclear lamin (T-47)	*Lamin*	76.0	nucleoskeletal/nucleus	antibody	10
Numb	*numb*	60.6	signaling/membrane	antibody	9
Oho-31	*oho31/Pendulin*	57.8	transport/nucleus, cytoplasm	antibody	8–9
Pan	*pangolin*	81.9	transcription factor/nucleus	antibody	9
p53	*p53*	43.7	transcription factor, tumor suppressor/nucleus	antibody	10
p55	*Chromatin assembly factor 1 subunit*	55.0	chromating remodeling, transcription/nucleus	antibody	10
p127	*lethal(2)giant larvae*	127.0	cytoskeletal and signaling, tumor suppressor/cell membrane	antibody	8
Rab11	*Rab-protein 11*	24.2	GTPase/endosome, trans-Golgi, cytoplasm	antibody	10
Ras2	*Ras oncogene at 64B*	22.2	GTPase/membrane	antibody	8
Rop	*Ras opposite*	68.0	transport/cytoplasm, membrane	antibody	9
Rpd3	*Rpd3*	58.3	histone deacetylase/nucleus	antibody	9
Rp21	*Ribosomal protein 21 M(3)80*	26.0	ribosomal protein/cytoplasm	antibody	9
Rp40	*stubarista*	30.2	ribosomal protein/cytoplasm, nucleus	antibody	8
Scribbled	*scribbled*	186.0	signaling/cell membrane	antibody	9
Sin3A	*Sin3A*	220.0	transcription, corepressor/nucleus	antibody	8
Smrter	*Smrter*	379.1	transcription/nucleus	antibody	8
α-Spectrin	*α-spectrin*	280.0	cytoskeletal/cell membrane	antibody	9
Taiman	*taiman*	215.0	transcription/nucleus	antibody	9
Trr	*trithorax-related*	260.0	histone methyltransferase/nucleus	antibody	8
α-Tubulin84B	*α-Tubulin at 84B*	49.9	cytoskeletal/cytoplasm	antibody	8
β-Tubulin56D	*β-Tubulin at 56D*	50.1	cytoskeletal/cytoplasm	antibody	8
Usp	*ultraspiracle*	54.0	nuclear receptor, transcription factor/nucleus	antibody	8
Wg	*wingless*	52.0	signaling/membrane, extracellular matrix	antibody	9

This table shows 47 proteins identified using laser confocal or fluorescence microscopy of antibody-stained salivary glands. Proteins are listed alphabetically with the corresponding gene name, molecular weight (in kDa), function and predominant cellular localization. The rightmost columns describe the detection method and predominant time of their release into lumen.

**Table 2:** List of proteins released by apocrine secretion and detected by fluorescent tagging.

Protein	Corresponding gene	MW (kDa)	Function/Cellular localization	Detection method	Time of release (hr APF)	Refe-rence
Asph	*Aspartyl β-hydroxylase*	89.8	oxidoreductase/endoplasmic reticulum	GFP	9	{a} *Flytrap ZCL1605
Atg5	*Autophagy-specific gene 5*	31.5	protein transport/cytoplasm	GFP	8–9	FBti 0131368
Atg8a (LC3)	*Autophagy-specific gene 8a*	14.4	autophagy ubiquitine-like/cytoplasm	GFP	8	FBti 0147141
α-subunit of Na^+^,K^+^-ATPase (Na^+^,K^+^-ATPase subunit alpha)	Atpalpha	100.0	ATPase/membrane	GFP	9	{a} *Flytrap ZCL2207
βTubulin56D GFP-βTub56D	*β-Tubulin at 56D*	51.0	cytoskeletal/cytoplasm	GFP	8	*Gavdos Protrap {b}
CG17324-Luciole	*CG17324*	59.9	UDP-glycosyltransferase	GFP	9	*Gavdos Protrap {b}
Chc	*Clathrin heavy chain*	191.2	transport/cytoplasm, membrane, vesicles	GFP	8	FBti 0115107
Clc	*Clathrin light chain*	23.8	transport/cytoplasm, membrane, vesicles	GFP	10	FBti 0027885
Clic	*Chloride intracellular channel*	30.2	ion binding/membrane	GFP	9	*Gavdos Protrap {b}
Cpn60	Heat shock protein 60	60.8	heat shock protein/mitochondrion	GFP	9	*Gavdos Protrap
Eb1	*Eb1*	32.5	microtubule-based process/microtubule associated complex	GFP	9	FBti 0141213
Hrb98DE	*Heterogeneous nuclear ribonucleoprotein at 98DE*	38.0	RNA processing/nucleus	GFP	9	{a} *Flytrap ZCL0588
Gilgamesh	*gilgamesh*	52.1	Ser/Thr-protein kinase/nucleus, membrane	GFP	8	*Gavdos Protrap {b}
Grasp65	*Grasp65*	47.7	transport/Golgi, endoplasmic reticulum	GFP	10	FBti 0040816
Histone 2A	*Histone H2A*	13.4	histone/nucleus	RFP	9	* {c} FBal 0285443
Ilk	*Integrin linked kinase*	50.7	kinase/membrane	GFP	8	{a} *Flytrap ZCL3192
Jupiter	*Jupiter*	22.3	cytoskeletal/nucleus, cytoplasm	GFP	9	*Gavdos Protrap {b}
Lac	*Lachesin*	39.9	structural/membrane	GFP	10	{a} *Flytrap G00044
Lamin C	*Lamin C*	69.9	nucleoskeletal/nucleus	GFP	10	{a} *Flytrap CB04957
Larp	*La related protein*	178.1	RNA binding/cytoplasm, nucleus	GFP	9	{a} *Flytrap YC0014
Moesin	*Moesin*	68.0	cytoskeletal, structural/membrane	GFP	8	* {d}
Pdi	*Protein disulfide isomerase*	55.8	protein folding/endoplasmic reticulum	GFP	9–10	FBti 0027861
Rbp1	*RNA-binding protein 1*	27.0	RNA processing/nucleus	GFP	8	*Gavdos Protrap {b}
RNA-3′-phosphate cyclase	*Rtc1*	42.1	RNA processing/nucleus, nucleolus	GFP	9	*Gavdos Protrap {b}
RNP 87F squid	*squid*	40.0	RNA binding/nucleus, cytoplasm	GFP	9	Gavdos Protrap {b}
Scribbler	*scribbler*	80.0	transcription corepressor/nucleus	GFP	9	*Gavdos Protrap {b}
Scyl	*scylla*	30.8	signaling/cytoplasm	GFP	8	FBti 0037939
Sgs3	*Salivary gland secretion 3*	32.2	extracellular glue/secreted	RFP	8	* {e}
Tcp-1eta	*Tcp-1eta*	59.4	chaperonin/cytoplasm	GFP	9	*Gavdos Protrap {b}
Tropomyosin 1	*Tropomyosin 1*	39.3	cytoskeletal/cytoplasm	GFP	9	FBti 0128132
VhaSFD	*Vacuolar H^+^-ATPase SFD subunit*	53.7	vATPase/vacuole	GFP	8–9	FBti 0027854
Zw3 Ser/Thr kinase	*shaggy*	56.0	protein kinase/cell junction, cytoplasm, nucleus	GFP	9	*Gavdos Protrap {b}

Table shows 32 proteins identified using GFP-/EYFP-/RFP-constructs, as mentioned also in Materials and Methods section. Also here proteins are listed alphabetically with the corresponding gene name, molecular weight (in kDa), function and predominant cellular localization. The rightmost columns describe not only the detection method but also predominant time of their release into lumen and whenever possible also genotype reference.

*non-FBti and non-FBal References related to Table 2 and 3.

{a} Flytrap (http://flytrap.med.yale.edu/).

Morin X, Daneman R, Zavortink M and Chia W (2001) A protein trap strategy to detect GFP-tagged proteins expressed from their endogenous loci in *Drosophila*. Proc. Natl. Acad. Sci. USA 98: 15050–15055.

{b} Gavdos Protein trap.

(http://biodev.obs-vlfr.fr/gavdos/protrap.htm) Alain Debec; Biologie du Développement, UMR 7009, CNRS/Université Pierre et Marie Curie, Observatoire Océanologique, Villefranche sur mer, 06230, France.

{c} Kanesaki T, Edwards CM, Schwarz US and Grosshans J (2011) Dynamic ordering of nuclei in syncytial embryos: a quantitative analysis of the role of cytoskeletal networks. Integr. Biol. (Camb.) 3: 1112–1119.

{d} Edwards KA, Demsky M, Montague RA, Weymouth N and Kiehart DP (1997) GFP-moesin illuminates actin cytoskeleton dynamics in living tissue and demonstrates cell shape changes during morphogenesis in *Drosophila*. Dev. Biol. 191: 103–117.

{e} Costantino BF, Bricker DK, Alexandre K, Shen K, Merriam JR, Antoniewski C, Callender JL, Henrich VC, Presente A and Andres AJ (2008) A novel ecdysone receptor mediates steroid-regulated developmental events during the mid-third instar of *Drosophila*. PLoS Genet. 4: e1000102.

**Table 3:** List of proteins released by apocrine secretion and detected by chromogenic staining.

Protein	Corresponding gene	MW (kDa)	Function/Cellular localization	Detection method	Time of release (hr APF)	Refe-rence
Antp	*Antennapedia*	43.0	transcription factor/nucleus	lacZ	9	
Arm	*armadillo*	93.0	cytoskeletal, signaling/membrane, cytoplasmic	lacZ	9	FBti 0018347
Brk	*brinker*	77.5	transcription factor/nucleus	lacZ	10	
Capt	*capulet*	45.6	actin binding/cytoplasm	lacZ	9	
CG14207 (Hsp20-like α-crystallin)	*CG14207*	20.8	heat shock protein/cytoplasm	lacZ	9	FBti 0038459
CG6175	*CG6175*	61.6	unknown	lacZ	8	
CG8668	*CG8668*	64.7	glycosyltransferase/Golgi, membrane	lacZ	8	
Cype	*cyclope*	8.3	cytochrome c oxidase/mitochondrion	lacZ	9	FBti 0005248
Dlc90F	*Dynein light chain 90F*	12.5	cytoskeletal/cytoplasm	lacZ	9	
Doa	*Darkener of apricot*	55.0	dual-specific protein kinase/cytoplasmic and nuclear	lacZ	8	FBti 0005439
DX16 (hn RBP1-like GFP-Ping)	*x16*	27.9	RNA processing/nucleus	lacZ	8	
Ec	*echinus*	188.4	ubiquitin thiolesterase,cytoplasm	lacZ	9	
En	*engrailed*	59.4	transcription factor/nucleus	lacZ	8	FBti 0002246
Fer2LCH	*Ferritin 2 light chain homologue*	25.2	iron binding/Golgi, secretory	lacZ	9	FBti 0005395
Fkh	*fork head*	54.0	transcription factor/nucleus	lacZ	8	
For	*foraging*	101.1	protein kinase/membrane	lacZ	9	FBti 0006974
Fray	*frayed*	60.3	PASK/SPAK kinase/cytoplasmic	lacZ	8	FBti 0005585
Int6	*Int6 homologue*	48.0	translation/cytoplasm	lacZ	9	
Lab	*labial*	67.5	transcription factor/nucleus	lacZ	8	FBti 0005424
LAMP1	*Lamp1*	34.8	vesicular/lysosome	lacZ	9	
Mod	*modulo*	60.3	DNA/RNA binding/nucleus, nucleolus, cytoplasm	lacZ	10	FBti 0009927
Ng-1	*new glue 1*	11.4	extracellular glue/secreted	lac Z	8	* {f}
Oda	*Ornithine decarboxylase antizyme*	28.3	enzyme inhibitor/cytoplasm	lacZ	9	
Pdcd4	*Programmed cell death 4 ortholog*	56.4	RNA metabolism/cytoplasm	lacZ	9	
Pnut	*peanut*	60.1	cytoskeletal, GTPase/membrane	lacZ	9	
Poly(A)-binding protein 2	*Pabp2*	33.0	RNA processing/nucleus, cytoplasm	lacZ	8	FBti 0071136
Puc	*puckered*	58.0	phosphatase/Golgi, endoplasmic reticulum	lacZ	8	
Pum	*pumilio*	156.0	translation/cytoplasm	lacZ	8	
RCC1-like protein	*Regulator of chromosome condensation 1 ortholog*	58.9	chromatin binding/nucleus	lacZ	10	
RhoGAP71E	*Rho GTPase activating protein at 71E*	66.4	signaling/membrane	lacZ	9	
RpS27A	*Ribosomal protein S27A*	17.9	ribosomal protein/cytoplasm	lacZ	9	FBti 0005278
Sktl	*skittles*	87.8	transferase/cell membrane, membrane	lacZ	8	
Sply	*Sphingosine-1-phosphate lyase*	60.3	decarboxylase/endoplasmic reticulum, membrane	lacZ	9	
Sra	*sarah*	31.4	signaling**/**cytoplasm, mitochondrion, nucleus	lacZ	10	
Syx13	*Syntaxin 13*	31.5	transport/membrane	lacZ	9	
Tau	*tau*	60.0	cytoskeletal/microtubule	lacZ	10	
Thor	*Thor*	12.9	translation/cytoplasm	lacZ	9	FBti 0009315
Tramtrack	*tramtrack*	97.0	transcription factor/nucleus	lacZ	8	FBti 0005154
Tropomyosin 1	*Tropomyosin 1*	39.3	cytoskeletal/cytoplasm, cytoskeleton	lacZ	10	
Twr	*twisted bristles roughened eye*	21.0	peptidase/membrane	lacZ	9	
vATPase subunit D	*Vacuolar H^+^ ATPase subunit 36*–*1*	27.6	vATPase/vacuole	lacZ	9–10	FBti 0006704
VhaSFD	*Vacuolar H^+^-ATPase SFD subunit*	53.7	vATPase/vacuole	lacZ	8–9	
Zw3 Ser/Thr kinase	*shaggy*	56.0	protein kinase/cell junction, cytoplasm, nucleus	lacZ	9	

Table 3 shows 44 entities detected by positive LacZ staining of *P*-element insertions, as described under Materials and Methods. Also these proteins are listed alphabetically with the corresponding gene name, molecular weight (in kDa), function and predominant cellular localization. The rightmost columns describe not only the detection method but also predominant time of their release into lumen and whenever possible also genotype reference.

*non-FBti and non-FBal References related to Table 2 and 3.

{f} Crispi S, Giordano E, D‘Avino PP, Peluso I and Furia M (2001) Functional analysis of regulatory elements controlling the expression of the ecdysone-regulated *Drosophila ng-1* gene. Mech. Dev. 100: 25–35.

The *lacZ*/*W* P-element insertion lines are listed in Table 3 and except *l(2)k07207* (*vATPase subunit D*) and *shaggy* (Istvan Kiss, Hungarian Academy of Sciences, Szeged), many of them were from Bloomington Stock Center.

### Protein and RNA synthesis

Total RNA synthesis in prepupal salivary glands was measured by incorporation of [5,6-^3^H]-uridine (30–60 Ci/mmol; Amersham/GE Healthcare Co.), essentially as described elsewhere [51]. Briefly, 20 pairs of salivary glands were dissected from 8/10–14 hr old prepupae, rinsed several times in PBS, transferred into 100 µl of Grace's medium diluted 5∶4 as described in Farkaš and Šuťáková [52] and supplemented with 20 μCi of [5,6-^3^H]-uridine and cultured for another 1 hr. Salivary glands were lysed in 20 mM Tris-HCl buffer pH 7.5 containing 1% SDS, 0.1% proteinase K, and 5 µl aliquots were TCA-precipitated on GF/A glass fiber filters (Whatman Ltd.), rinsed 3 times with each 20 ml of 15% and 8% TCA, and 20 ml of ethanol. After drying, radioactivity captured on filters was measured in LKB 1217 RackBeta or Beckman 6500 liquid scintillation counters.

Protein synthesis was monitored by incorporation of ^35^S-methionine (1200 Ci/mmol; Amersham/GE Healthcare Co.) or ^3^H-leucine (NEN; 160–200 Ci/mmol) into *in vitro* cultured glands dissected from prepupae at particular times, as described previously [51]. Briefly, 10 pairs of salivary glands were dissected from 10–12 hr old prepupae, rinsed several times in PBS, transferred into 100 µl of Grace's medium diluted 5∶4 as described in Farkaš and Šuťáková [52] and supplemented with 50–100 μCi of ^35^S-methionine or 10 μCi of [4,5-^3^H]-leucine and cultured for another 1 hr. Salivary glands were then extracted in Tris-HCl buffer pH 6.8 containing 10% glycerol, 1% mercaptoethanol and 2% SDS at 100°C for 5 min. One µl aliquots in duplicates were taken for TCA precipitation, and filtered through GF/C glass fiber filters (Whatman Ltd.) on Hoefer 10-manifold filtration unit, rinsed 3 times with 20 ml each of 15% TCA, 8% TCA, and ethanol. After drying, radioactivity captured on filters was measured in LKB 1217 RackBeta or Beckman 6500 liquid scintillation counters.

Proteins were analyzed by SDS-polyacrylamide gel electrophoresis (SDS-PAGE) in a discontinuous pH gradient according to Laemmli [53] employing a 10% separating gel. The proteins were visualized by staining with Coomassie Brilliant Blue R-250 [54] or ammoniacal silver nitrate [55]. Radiolabelled proteins were detected by fluorography as described by Laskey and Mills [56].

For RNA and protein synthesis, salivary glands were intentionally dissected and cultured *in vitro* to exclude the possibility that macromolecules synthesized by other tissues or in the haemocoel would be taken up by salivary gland cells from the haemolymph.

### Immunocytochemistry and confocal microscopy

Salivary glands were dissected while viewed using a stereomicroscope in Ringer's solution and fixed in Pipes-buffered 4% paraformaldehyde (pH 7.2). In order to stain tissue with antibodies they were permeabilized with 0.1% Triton X-100 in PBS (PT) and then blocked with PT containing 2% fraction V of bovine serum albumin (PBT) and 2% goat serum. After blocking, the tissues were incubated overnight at 4°C with primary antibodies: rabbit anti-p127, rabbit anti-Rab11, rabbit anti-Rop, rabbit anti-Ras2, rabbit anti-myosin II, as well as mouse anti-myosin II, mouse anti-β-tubulin, mouse anti-BR-C, mouse anti-lamin T47, mouse anti-EcR, mouse anti-Syntaxin 1A, guinea pig anti-Scrib, rabbit anti-Doa, rabbit anti-Rpd3, rabbit anti-Sin3A, rabbit anti-p55, mouse anti-E74, mouse anti-E75, mouse anti-Usp, mouse anti-Arm, rabbit-anti-Met, mouse anti-En, mouse anti-Wg, rabbit anti-Oho31, rabbit anti-Rp21, rabbit anti-Rp40, rabbit anti-FTZ-F1β, rabbit anti-Taiman, rabbit anti-Smrter, mouse anti-p53, rabbit anti-KrH, mouse anti-α-Spectrin, mouse anti-fibrillarin, human anti-PDH, rabbit anti-ME, *etc*. (for more details see Table 1). To detect the primary antibodies, FITC-conjugated anti-guinea pig, Cy3-conjugated anti-rabbit and Cy5-conjugated anti-mouse affinity purified F(ab’)_2_ specific pre-absorbed secondary antibodies were used (Jackson ImmunoResearch Laboratories, Inc.) diluted 1∶200. F-actin was detected using AlexaFluor_488_- or AlexaFluor_546_-phalloidin (Molecular Probes Inc.) at 0.04 nM concentration. Depending on the fluorochrome combination for antibodies and phalloidin, nuclei were counterstained for DNA either with 5 µg/ml Hoechst-33258 (Calbiochem), 0.5 µg/ml Oli-Green or 0.1 µg/ml Toto-3 (both Molecular Probes Inc.). After extensive destaining in PT solution, tissues were mounted in Elvanol and scanned on Zeiss LSM-410, LSM-510 Meta or Leica TCS SP5 laser confocal microscopes using 40× (oil NA 1.4) lenses. The RGB-bitmap images obtained were processed using Zeiss AIM LSM5 software and Adobe PhotoShop, and assembled into figure plates using Aldus FreeHand or Adobe PhotoShop applications.

To detect living mitochondria, dissected salivary glands were loaded with laser dye Rhodamine 123 (Kodak) at 15 µM concentration in Grace's medium diluted 5∶4 as described elsewhere [52] for 10 min. After 3 extensive washes in fresh Grace's medium, living glands were examined at 488 nm in a drop of diluted Grace's medium using Leica DMR-B fluorescence, Olympus IX70 fluorescence or a Zeiss LSM-510 Meta laser confocal microscopes.

The secretion of GFP-, EYFP- or RFP-fusion proteins (coming from GFP-tagged gene disruption and fly-trap projects provided by A. Debec, W. Chia, H. Bellen, A. Spradling, G.M. Rubin, and via the Bloomington Drosophila Stock Center) was monitored *in vivo* after dissection of 8–10 hr old prepupal salivary glands in a 30 µl drop of Grace's medium at the appropriate wavelength under Leica DMR-B fluorescence, Olympus Provis AX70 or Zeiss LSM-510 Meta laser confocal microscopes.

### X-Gal staining

For chromogenic detection of β-galactosidase (*lacZ*) expression in *P-element* strains, tissues were fixed in 5% glutaraldehyde in PME (Pipes-MgSO_4_-EGTA) buffer, pH 7.2, permeabilized with 0.2% Triton X-100 in PME (PMET) and incubated in a 6.1 mM potassium ferrocyanide/ferricyanide solution containing 0.2% 5-bromo-4-chloro-3-indolyl-β-D-galactopyranoside (X-Gal) at room temperature according to Bellen *et al*. [57], as modified by Kobayashi and Okada [58]. After the desired level of staining was obtained, tissues were extensively washed in PMET to remove excess X-Gal and mounted in glycerol or Elvanol. Stained tissues were imaged using Nikon Microphot-FXA or Leitz Aristoplan microscopes equipped with a cooled digital camera (Spot Instruments Inc.).

### *In situ* hybridization

The nuclear genomic or mitochondrial DNA/RNA were detected by non-radioactive *in situ* hybridization to paraformaldehyde-fixed prepupal salivary glands [59]. Briefly, to unambiguously detect mtDNA, a 220 bp-long segment of *Drosophila* mtDNA corresponding to nucleotides 2580 through 2800 of the mitochondrial DNA from GenBank J01404 [60], [61] encompassing three genes (including the 3'-OH end of mt cytochrome c oxidase I, the entire coding sequence of mt tRNA-Leu, and the 5'-OH end of mt cytochrome c oxidase II) was PCR amplified using a *Taq* and *Tgo* DNA polymerase blend from the High Fidelity Master Mix II kit (Roche) and cloned into *Eco* RI/*Not* I sites of pBS II KS vector (Stratagene). To detect nuclear genomic DNA, we used a cDNA clone for *Doa*, a gene encoding the dual-specific LAMMER kinase cloned into pBS II KS vector [62]. A linearized plasmid (0.5 µg) was diluted in 50 mM Tris-HCl, 10 mM MgCl_2_ and 10 µM dithioerythritol supplemented with hexanucleotide mix, 3 dNTPs and digoxygenin-conjugated dUTP, and the probe was generated after addition of 2 units of Klenow enzyme of the DNA polymerase I (Roche) for 6 hr at 37°C according to the manufacturer instructions. The DIG-labeled probe was pre-heated at 65°C and then hybridized to DNase-free RNase-treated (Roche) salivary gland tissue at 37°C for 16 hr. The hybridized probe was subsequently detected either using anti-DIG-alkaline phosphatase conjugated sheep IgG (Fab fragments) secondary antibody using NBT/BCIP chromogenic substrates (Sigma) or anti-DIG-FITC conjugated sheep IgG (Fab fragments) secondary antibody (Roche or Jackson IR Labs). In some cases tissue was counterstained with 0.04 nM AlexaFluor_546_-phalloidin (Molecular Probes Inc.) and 5 µg/ml Hoechst-33258 (Calbiochem) to detect actin and DNA, respectively. After extensive washing, salivary glands were finally mounted in Elvanol and examined under light or laser confocal microscope as above.

### Western blotting

Ten pairs of prepupal salivary glands from animals 8–10 hr APF were dissected and transferred to a fresh 10 µl drop of Ringer‘s containing a protease inhibitors cocktail (1 mM bestatin, 100 µM chymostatin, 7.5 µM antipain, 1 µM leupeptin, 50 µg/ml AEBSF, 1 mM phenylmethylsulfonylfluorid, 1 µM aprotinin, 10 µM benzamidine, 8 µM phosphoramidone and 20 µg/ml E64; components from Calbiochem, Roche and Sigma). Each salivary gland was carefully and gently squeezed along its longitudinal axis with a No. 5 Dumont extrafine or Moria superfine tweezers to make gentle pressure that would expel the luminal contents into the Ringer drop without injuring the gland cells as described below. Ten pairs of late 3^rd^ instar larval or early prepupal glands were used as controls, and extracted as entire organs. The Ringer‘s drop with the secreted material from 8–10 hr prepupal glands was immediately transferred to a clean eppendorf tube and 10 µl SDS-sample buffer (12.5 mM Tris-HCl, 2% SDS, 5% β-mercaptoethanol, 10% glycerol pH 6.8 plus protease inhibitors cocktail) added. The tube was heated for 5 min at 100°C, centrifuged at 16,000×g for 15 min and the supernatant frozen at −80°C. The same extraction procedure was applied also to late larval and early prepupal glands. Protein extracts were loaded on 10% polyacrylamide-SDS gel and electrophoresed at a constant current of 20 mA for ∼3 hr or until the dye front of the samples reached bottom of the gel. Separated polypeptides were transferred to Immobilon-P PVDF membrane (Millipore) using a semi-dry blot apparatus (Bio-Rad), and proteins were detected using anti-Rab11, anti-BR-C, anti-p127, anti-lamin primary antibodies (specifications see above), followed by alkaline phosphatase-conjugated secondary antibodies (Sigma). Protein bands were visualized using CSPD/Nitroblock chemiluminescence substrates for alkaline phosphatase (ABI-Tropix Inc.) and membrane exposed to X-ray film (Fuji Ltd.).

### Proteomic analysis

#### Sample collection and electrophoresis

Twenty pairs of prepupal salivary glands were dissected from animals 8–10 hr APF and transferred to a fresh 10 µl drop of Ringer (diluted 1∶1) containing the protease inhibitors cocktail (1 mM bestatin, 100 µM chymostatin, 7.5 µM antipain, 1 µM leupeptin, 50 µg/ml AEBSF, 1 mM phenylmethylsulfonylfluorid, 1 µM aprotinin, 10 µM benzamidine, 8 µM phosphoramidone and 20 µg/ml E64; components from Calbiochem, Roche and Sigma). Each salivary gland was carefully and gently squeezed along its longitudinal axis with a No. 5 Dumont extrafine or Moria superfine tweezers to use delicate pressure to expel the luminal contents into the Ringer drop without injuring gland cells. Making the Ringer's slightly hypotonic facilitated the release of the lumen contents into the drop. This process could be easily monitored using a good stereomicroscope (Leica MZ9.5 or MZ12) with adjustable bright field transillumination (so-called Wild M5A or M420 „Durchlichtstative“ base). The treated gland was immediately removed from the drop and processed separately for protein extraction. After the luminal contents of all 20 pairs of glands were pressed out, the Ringer‘s drop with the secreted material was immediately transferred to a clean eppendorf tube and 10 µl of SDS-sample buffer (12.5 mM Tris-HCl, 2% SDS, 5% β-mercaptoethanol, 10% glycerol pH 6.8 plus protease inhibitors cocktail) added. The sample was extracted for 5 min at 100°C, centrifuged at 16,000×g for 15 min and the supernatant frozen at −80°C. During these and all subsequent steps, extreme care was taken to avoid any air-born contamination of the samples (dust, bacteria, human skin *etc*.). Upon thawing, protein extracts from 200 gland pairs (10 independent extractions of 20 pairs) were quickly pooled and loaded onto a 10% polyacrylamide-SDS gel and electrophoresed at a constant current of 20 mA for ∼3 hr or until front of the samples reached bottom of the gel. The separated proteins in the gel were fixed in 50% methanol and 10% acetic acid for 1 hr and visualized with Coomassie brilliant blue R-250 (Serva), or PageBlue protein stain (Fermentas).

#### Enzymatic in-gel digestion and chemical derivatization

Stained and dissected protein bands were processed using standard protocol for mass spectrometry protein identification according to Shevchenko *et al*. [63] with minor modifications. Briefly, the cut gel pieces containing separated proteins were washed by addition of 100 µl of 100 mM NH_4_HCO_3_ and 400 µl acetonitrile (5 min). The washing solution was removed and the proteins were in-gel reduced with 50 µl of 10 mM DTT in 100 mM NH_4_HCO_3_ (30 min at 56°C). After addition of 400 µl acetonitrile and brief vortexing, the supernatant was removed. The proteins were alkylated in-gel with 50 µl of 50 mM iodoacetamide in 100 mM NH_4_HCO_3_ (30 min) in the dark. The alkylation reaction was stopped by removing the reaction solution and by washing gel pieces with 400 µl of 100 mM NH_4_HCO_3_ (5 min) followed by addition of 400 µl acetonitrile (5 min). The shrunk gel pieces were first rehydrated at 4°C (2 hr) in 10 mM NH_4_HCO_3_, then digested with 1 mM sequencing-grade gold trypsin (Promega) in 10 mM NH_4_HCO_3_ at 37°C for 12 hr and subsequently acidified with 5% formic acid. The extracts were dried down using an Eppendorf 5301 centrifugal vacuum concentrator at 30°C. The recovered peptides were dissolved in 50 µl of 0.1% TFA. Further purification was achieved by C_18_ ZipTip pipette tips (Millipore, Bedford, MA, USA) used according to manufacturer's instructions.

#### MALDI-TOF/TOF Mass Spectrometry

MALDI-TOF/TOF mass spectrometry measurements were performed using 4800 Proteomics Analyzer (Applied Biosystems, Framingham, USA). The MS and MS/MS data were acquired and processed using 4000 Series Explorer v.3.6 (Applied Biosystems). Up to 10 precursors from the MS spectra with S/N ratio of greater than 100 were selected from particular sample spot analysis for the MS/MS fragmentation analysis and acquisition, and sorted according to the decreasing S/N value; the contaminant peaks (keratins, trypsin autolysis, *etc*.) were automatically excluded from the MS/MS analysis within the interpretation method of the 4000 Series Explorer software. The isolation parameter for precursor selection was set at 200 for the resolution of ion gating mechanism. The stainless steel target with 384 sample spots (with additional 13 calibration spots) and α-cyano-4-hydroxycinnamic acid (5 mg/ml) as MALDI matrix in 60% acetonitrile/0.1% TFA (v/v) were used in all MALDI experiments. Digests were purified either using stop-and-go extraction tips [64] with subsequent addition of MALDI matrix to the sample spot containing eluted peptides or using a matrix-tip with direct elution of peptides and MALDI matrix on the MALDI target plate [65]. The accelerating voltage in the ion source for the MS mode was 20 kV. In the MS/MS mode, the accelerating voltage was 8 kV, which was after ion selection modified that ions passing collision cell posses 1 keV of kinetic energy and after ions passed the collision cell the voltage raised to 15 kV. Delayed extraction was applied in all experiments and it was optimized for m/z 2100 in the MS mode. This MALDI-TOF/TOF instrument is equipped with an Nd-YAG laser at 355 nm producing 3–7 ns pulses with a 200-Hz firing rate. The maximum pulse energy was 20 µJ and it was attenuated appropriately for the analysis of the samples. Both MS and MS/MS analyses in the positive mode were performed using reflectron. The dual microchannel plate detector was set for 1.94 kV in the MS mode and 2.16 kV in the MS/MS mode. The peaks were detected using the internal algorithm of the 4000 Series Explorer software with parameter S/N set for 10 in the MS mode and 5 in the MS/MS mode using the cluster area optimization feature.

#### Protein identification

The peak lists in the Mascot generic format were generated from mass spectra using the Peaks-to-Mascot function incorporated in the 4000 Series Explorer software. The peaks from the MS analysis were detected in an m/z range of 700–5000 with an S/N ratio greater than 18, whereas the MS/MS peaks with S/N ratio greater than 9 were detected in the range from m/z 68 up to an m/z value of 50 m/z units lower than precursor m/z value. These peak lists contained both MS information from the MS run and also information from MS/MS run about fragmentation data of selected precursors; they were then submitted through Mascot Daemon software (ver. 2.1.0) to the Mascot database search engine (local installation, ver. 2.1.04). The following parameters were used for the combined search (MS and MS/MS data): database - UniProt/Swiss-Prot (ver. 2011_11 - Nov 16, 2011) or NCBInr (ver. Nov 27, 2011); taxonomy - all entries (number of sequences: 12603350); enzyme - trypsin; allowed missed cleavages - 1; fixed modifications - carbamidomethyl (C); variable modifications - oxidation (M), pyro-carbamidomethyl (N-term C), pyro-Glu (N-term E), pyro-Glu (N-term Q); peptide tolerance - 50 ppm; MS/MS tolerance - 300 mmu; peptide charge - (+1); monoisotopic masses; instrument - MALDI-TOF-PSD. Hits obtained with a probability lower than 0.05 to be a randomly occurring match and also providing at least one successful peptide fragmentation confirming the identity of the protein were considered as successful protein identifications.

### Transmission electron microscopy (TEM)

Upon dissection, salivary glands were immediately fixed in 2% glutaraldehyde +4% formaldehyde (PolySciences Europe GmbH., Eppelheim, Germany) in 0.1 M cacodylate buffer containing 0.25 M sucrose (pH 7.2) for 2 hr at room temperature, postfixed in 1% osmium tetroxide (Serva Feinbiochemica GmbH., Heidelberg, Germany) in 0.1 M cacodylate buffer, dehydrated in ascending series of ethanol, infiltrated in propylene oxide, and embedded in Durcupan ACM resin (Fluka AG, Buchs, Switzerland) according to Kushida [66], [67] as modified by Glauert [68] and Mráz *et al*. [69]. Durcupan serial sections were made transverse to the longitudinal axis of the gland, beginning from the most posterior end and extending anteriorly through the mid region. Ultrathin sections made on Reichert-Jung/Leica Ultracut ultramicrotomes equipped with diamond knife were contrasted with uranyl acetate [70] and lead citrate [71], [72] with modifications of Mazza *et al*. [73]. Electron micrographs were collected by a Jeol 100 CX electron microscope operating at 60 kV and Tecnai Sphera G2 electron microscope operating at 80 kV.

### Scanning electron microscopy (SEM)

Immediately after dissection salivary glands were fixed in 2% glutaraldehyde +4% paraformaldehyde (PolySciences Europe GmbH., Eppelheim, Germany) in 0.1 M cacodylate buffer containing 0.25 M sucrose (pH 7.2) for 20 min at room temperature, rinsed and postfixed in 1% osmium tetroxide (Serva Feinbiochemica GmbH., Heidelberg, Germany) in 0.1 M cacodylate buffer for at least 2 hr. Salivary glands were dehydrated gradually in 30%, 50%, 70%, 96% and 100% ethanol. Dehydration in 100% ethanol was done at least twice and then exchanged for 100% acetone followed by a acetone:hexamethyldisilazane (HMDS) mixture (1∶1). Finally, glands were treated with HMDS (Sigma) for 20 to 30 min and air dried under a clean dust-free environment as described by Beňo *et al*. [74]. HMDS was used here in place of critical point drying in way similar to Peldri II [75 ver. 2011_11 - Nov 16, 201177]. Salivary glands were cemented on aluminum or stainless steel stubs with Scotch double-sided tape or carbon conductive tape (Electron Microscopy Sciences Inc. or Agar Scientific Ltd.) and covered by gold-palladium alloy using a Balzers SCD-030 sputter coater. Samples were viewed and photographed in a Hitachi S-800 ultra-high resolution scanning electron microscope with a field emission electron source operating at 10 or 15 kV.

## Results

### Protein extrusion in late prepupal salivary glands is an apocrine secretion

**Figure 1:**
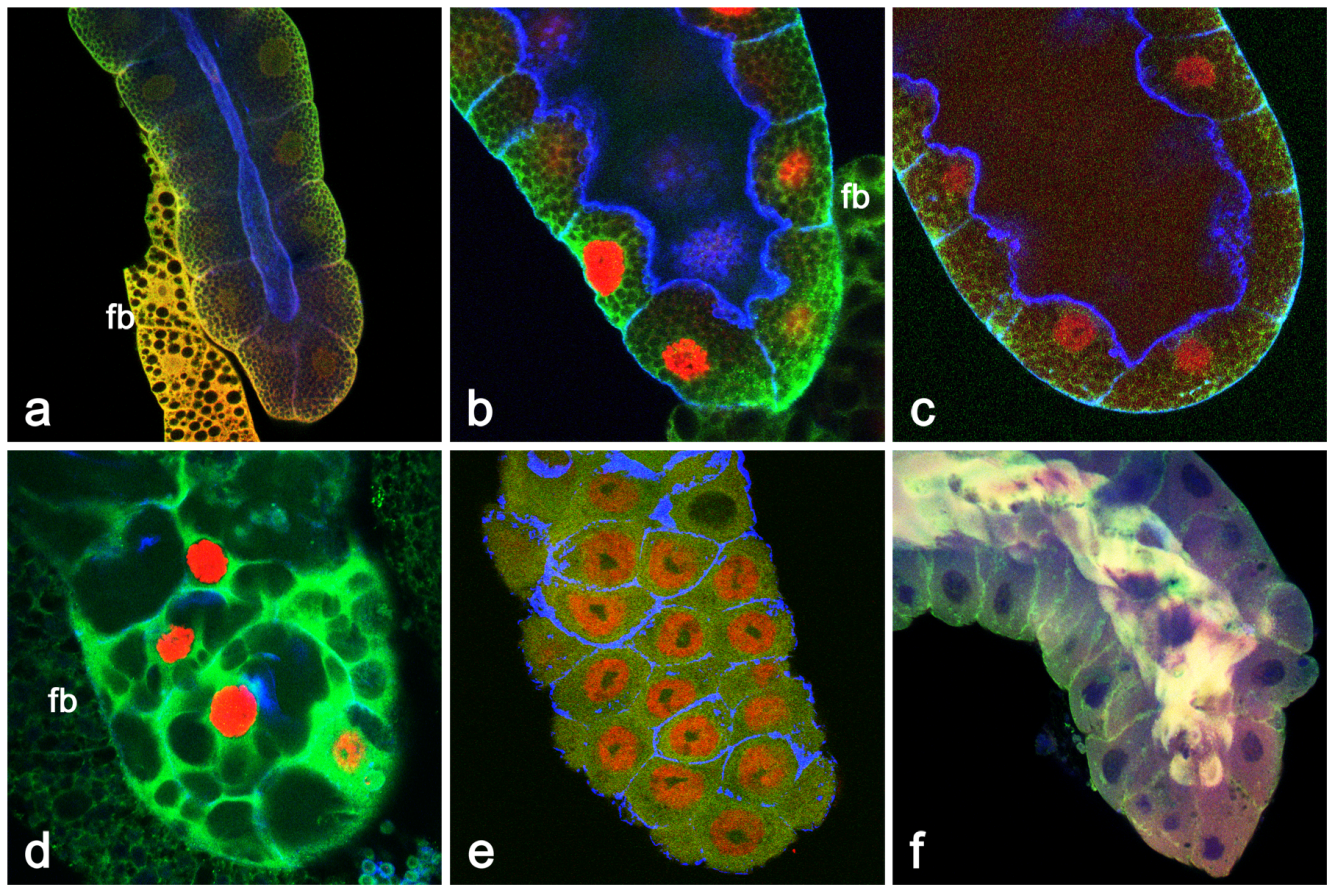
The course of major developmental events in the late larval (in late 3^rd^ instar larva) and prepupal salivary glands illustrated by staining with antibodies to highlight appropriate structures. (**a**) At -12 hr prior to pupariation, when Sgs glue proteins and secretory granules are synthesized, a dense “reticulate”meshwork forms from cytoskeletal components inside cells; myosin II (red), p127^l(2)gl^ (green) and filamentous actin (blue). (**b**) During metamorphic pulse of ecdysteroids at 7 hrs prior to pupariation (-7 hr), the larval salivary glands start to release the accumulated secretory granules into the lumen by exocytosis; transcription factor BR-C (red), p127^l(2)gl^ (green) and filamentous actin (blue). (**c**) At -3 hr prior to pupariation (-3 hr), exocytosis is complete and the salivary gland undergoes glue solvatation, increasing the diameter of the lumen. This solvatation will facilitate the expectoration of the glue at the pupariation; myosin II (red), p127^l(2)gl^ (green) and filamentous actin (blue). (**d**) About +2 hr APF, the salivary gland cells become highly vacuolized by membrane recycling due to massive endocytosis, a consequence of exocytosis; BR-C (red), p127^l(2)gl^ (green) and filamentous actin (blue). (**e**) The process of vacuolization and membrane recycling is consolidated by +7 hr APF, shortly prior to the next secretion; BR-C (red), p127^l(2)gl^ (green) and filamentous actin (blue). (**f**) At +8 hr APF, the salivary glands are showing an early phase of release of myosin II, p127^l(2)gl^ and filamentous actin into the centrally located lumen. fb in (**a**), (**b**), (**d**)  =  piece of adherent fat body. All confocal images 400×.

During a study where we attempted to make a detailed temporal description of the events prior to PCD in the *Drosophila* salivary glands [47], we observed a previously overlooked process of massive protein extrusion about 8 to 10 hr after pupariation (APF). As illustrated in Figure 1, the salivary glands in the late 3^rd^ instar larvae accumulate secretory glue granules (a) which start to be released by exocytosis into the centrally located lumen following an ecdysone pulse about 5 to 6 hr prior to pupariation (b). During the next two to three hr the secreted glue becomes liquified by the solute taken from the hemolymph resulting in the wide lumen (c). During the first hours after pupariation and glue expectoration, the salivary gland cells become vacuolized by enormous amounts of endocytosis (d). Within 6 to 7 hr after puparium formation (APF), the vacuoles are consolidated by continued endosomal trafficking towards ER and Golgi (e). Figure 1f shows that proteins detected by specific antibodies become released into centrally located gland lumen during the eighth hour of prepupal development, and that this process continues for the next ∼2 hr.

**Figure 2:**
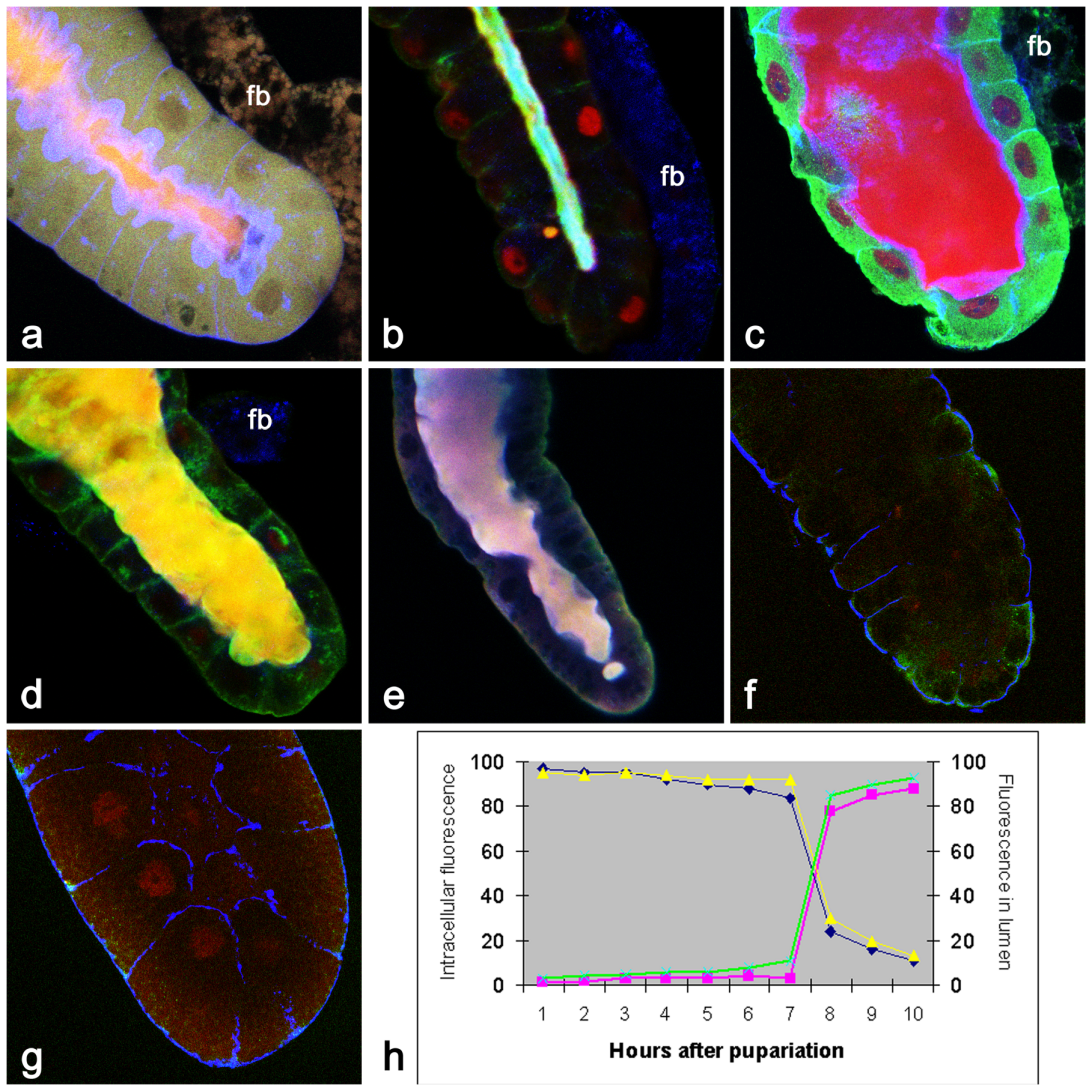
Immunological evidence for massive release of proteins in the salivary glands of 8–10 hr old prepupae. (**a**) +8 hr APF: There is an early phase of release of myosin II (red) and β-tubulin (green), while filamentous actin(blue) has become highly accumulated at the apical membrane; (**b**) +8.5 hr APF: Although α-catenin (blue) and nuclear Smrter (green) have already been completely released into lumen, transcription factor BR-C is still present in some nuclei (red). (**c**) +9 hr APF: At the mid-phase of secretion, BR-C (red) is mostly released into the wider lumen while cytosolic Rop (green) and filamentous actin (blue) are still mostly retained at their normal cellular locations. (**d**) +9.5 hr APF: The entire immunohistochemically detectable pool of filamentous actin (blue), myosin II (green) and nuclear Rpd3 (red) become visible only in the lumen. (**e**) +10 hr APF: The previously released filamentous actin (blue) become undetectable, and ribosomal protein Rp21 (green) and nuclear receptor EcR (red) are solely detected in the lumen. (**f**) +10.5 hr APF: The lumen has been emptied, and filamentous actin (blue) starts to be detected again only on basal surface. Although the salivary gland was stained also for the presence of Rop (green) and transcription factor BR-C (red), these proteins were not detected. (**g**) +11 hr APF: by this time, in addition to filamentous actin (blue) being visible on the basolateral membranes and slightly detectable at the apical surface, BR-C (red) begins to be detected again in nuclei. We speculate that th e low red cytoplasmic signal could represent freshly synthesized BR-C prior to its being imported in nuclei. However, Rop (green) is not yet detected by this time. fb in (**a**), (**b**), (**c**), (**d**)  =  piece of adherent fat body. Described massive protein secretion is accompanied by the relocation of measurable fluorescence from salivary gland cells to the extracellular gland lumen during 8 to 10 hr APF (**h**). The intracellular *vs*. lumenal distribution of representative proteins (p127: blue (intracellular) *vs*. green (lumenal), β-tubulin: yellow (intracellular) *vs*. magenta (lumenal)) was quantified by measuring the fluorescence signal [Cy5 (633 nm) for β-tubulin; Cy3 (546 nm) for p127; fluorescence intensity was evaluated by using Histogram module of Zeiss AIM LSM5 application] associated with a protein at hourly intervals following pupariation from each of 5 independent glands. All confocal images 400×.

Depending on the phase of this secretion and the type of protein secreted (detected by antibodies), one can observe differential release of proteins in time. For example, stronger accumulation of filamentous actin at apical membrane, even though non-muscle myosin II and β-tubulin are being released in the lumen during the first hour of the secretory process (Figure 2a). While some proteins such as α-catenin and nuclear Smrter, the EcR-coupled transcriptional corepressor, are released almost completely during the first hour of secretion, the transcription factor BR-C stays in nuclei (Figure 2b). During the more advanced phase of the protein extrusion (9^th^ hr APF), when the lumen is at its widest, it become filled with ecdysone-regulated transcription factor BR-C (red) while cytoplasmic Rop (green) is still retained in the cytoplasm (Figure 2c). By this time, nuclear histone deacetylase Rpd3 along with myosin II are both present in the lumen (Figure 2d). During the tenth hour APF, any remaining nuclear receptor EcR (red) and ribosomal protein P21 (green) as well as filamentous actin (blue) are all released into lumen (Figure 2e). As a consequence of this massive extrusion, by the end of the tenth hr APF, the signal of many intracellular proteins as detected by antibodies becomes weaker or undetectable (Figure 2f). However, some proteins at +11 hr APF can be detected, at least in modest amounts, at their original sites again (Figure 2g), indicating that the entire pool of cell proteinaceous components was not released, or alternatively, that they were quickly replaced by newly synthesized proteins. In summary, this massive protein secretion corresponds with relocation of measurable fluorescence signal from salivary gland cells to the extracellular gland lumen (Figure 2g).

**Figure 3:**
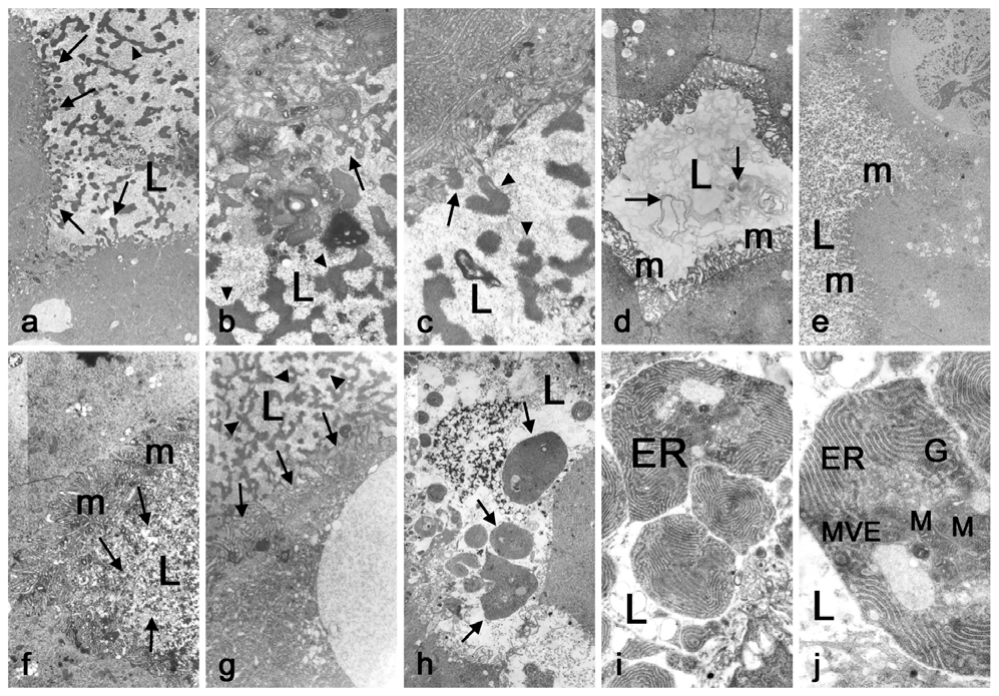
Transmission electron microscopy reveals an apocrine process in 8–10 hr old prepupal salivary glands. (**a**) *Prima vista* evidence of apocrine secretion is documented by apical protrusions (arrows) and numerous cytoplasmic fragments (arrowheads) inside lumen of the salivary glands from +9 hr APF animal; 2700×. Higher magnification views (**b** and **c**) of the apocrine process showing details of electron-dense material (arrows) released from the apical surface (arrowheads) of 9-hr old prepupal salivary gland cells; 8000× and 10000×, respectively. However, at the very early phases of apocrine secretion, +8 hour APF, the salivary gland cells show prominent and numerous microvilli (m) and the lumen is filled with “uncertain” whorled membraneous-like (arrows) (**d**) or electron-translucent filament-like material (**e**); both 2700×. Slightly later (+8.5 hr APF), the apical surface of the cells still contains numerous microvilli (m), but the material inside the lumen becomes electron dense and almost evenly distributed (arrows), consisting of many small pieces (**f**); 4000×. At the mid-phase of apocrine secretion (+9 hr APF), microvilli (m) are less abundant (arrows), and larger pieces and more electron dense material (arrowheads) start to appear in the lumen (**g**); 6700×. At later stages of apocrine secretion (+10 hr APF), the microvilli are absent and the luminal material becomes flocculent; it stays electron-dense, and larger pieces of material (arrows) are irregularly scattered in the lumen. Some of these clearly contain structured material of the cytoplasm including ER, Golgi (G), mitochondria (M) or multivesiculated elements (MVE) (**h**, **i**, **j**); 2700×, 8000× and 14000×, respectively. L in all images means lumen.

Since no secretory vesicles were observed, and no fluorescently-detectable increased Golgi zone areas or other exocytosis-associated activity could be observed, we decided to use transmission electron microscopy to verify that this massive protein extrusion was not being achieved by exocytosis. Indeed, EM images of the extrusion process in 8 to 10 hr old prepupal glands not only confirmed that proteins are not released by exocytosis but indicated that the process has typical attributes of apocrine secretion that entails the loss of part of the cytoplasm including apical protrusions and cytoplasmic fragments inside the lumen of the glands. These cytoplasmic fragments contain various types of electron-dense material such as small pieces of membranes, free ribosomes, endoplasmic reticulum *etc*. (Figure 3a throughout d). At the very early phases of apocrine secretion, during the eighth hour APF, the salivary gland cells show prominent and numerous microvilli and their lumen is filled with “uncertain” whorling membranous-like (Figure 3d) or electron-lucent filament-like material (Figure 3e). Slightly later, the apical surface of the cells still contains plenty of microvilli, and the material inside the lumen becomes electron dense and almost evenly distributed, consisting of many small pieces (Figure 3f). At the mid phase (+9hr APF), microvilli are present but less abundant, while larger pieces of more electron dense and compacted material start to appear in the lumen (Figure 3g). At the later stages of secretion, the microvilli are almost absent and the luminal material becomes flocculent. It is electron-dense, irregularly scattered in the lumen in the form of larger pieces, some of which clearly contains structured material of the cytoplasm including ER, Golgi or mitochondria *etc* (Figure 3h, i, j).

**Figure 4:**
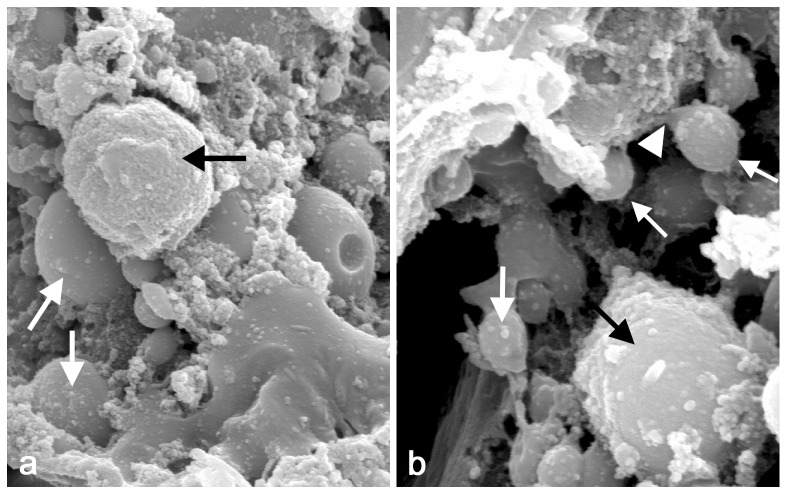
Scanning electron microscopic images of the apocrine process in the 9 The gland, dissected under the stereomicroscope and having a lumen evidently filled with material, was fixed and processed to critical point drying, after which it was broken up to expose inferior portion that included the luminal surface, and then sputter coated. The image reveals (**a**) numerous aposome-like spheres (arrows) and various material-bearing structures on the surface of apical membrane (10000×). In addition, at higher magnification (**b**), some of these spheroid structures (arrows) displayed constrictions and show a decapitation of the aposome's stalk (arrowheads) (20000×).

Numerous papers and reviews dealing with apocrine secretion in mammals (for review, see Gesase and Satoh, 2003 [26]) report that apical protrusions are released by a pinching-off process or the gradual constriction and decapitation of the stalk of an aposome. This process was not clearly recognizable in *Drosophila* salivary glands using TEM. Therefore, in order to assess this possibility we employed scanning electron microscopy (SEM) in 8–10 hr prepupal salivary glands. Using this approach, we identified the presence of numerous aposome-like structures on the apical membrane surface of the gland lumen, some of which displayed constriction and decapitation of the stalk of an aposome (Figure 4a, b). This further certified that the massive protein secretion occurs via an apocrine process.

### Apocrine secretion is not selective to protein categories

One of the fundamental questions about this newly discovered apocrine secretion in the *Drosophila* salivary glands was what kind of proteins it releases and whether the secreted material contains any specific proteins that could help shed light on the process' physiological significance. We used two approaches to address these questions: immunohistochemical detection at the light microscope level of extruded proteins and top-down proteomic identification of components isolated from the secretion. For the former, we used a panel of antibodies available in our laboratories or antibodies that were readily available from colleagues. We also randomly selected several LacZ- and GFP-protein trap transgenic fly stocks available in *Drosophila* research community, known to be expressed either ubiquitously or strongly in the salivary glands, and assessed whether LacZ or GFP signal was present in the lumen of 8–10 hr old prepupae.

For the proteomic analysis we collected multiple samples each containing the secretion released into the lumen of prepupal glands from at least 200 independent gland pairs. The pooled samples were separated by 1-dimensional electrophoresis, and individual fractions isolated from the gel were reduced, alkylated, trypsin-digested, chromatographically separated and their proteins identified by MALDI-TOF/TOF mass spectrometry.

**Figure 5:**
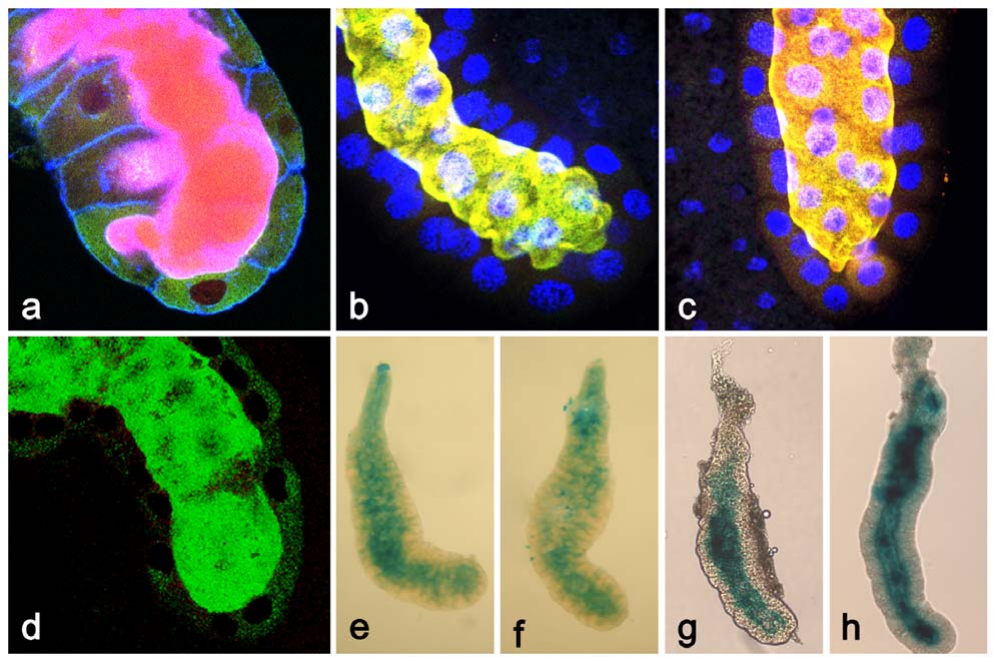
A great variety of proteins are detected by antibody, GFP-/EYFP-/RFP-fusion constructs, or X-Gal staining for active β-galactosidase produced by *lacZ*-containing *P*-element insertion stocks. We consistently used 9–10 hr old prepupal salivary glands for these types of detection. (**a**) Salivary gland showing the presence of nuclear receptor E75 (red) and a portion of the cytoplasmic signaling protein Ras2 (green) in the lumen. The cortical membrane is stained with AF_488_-phalloidin for F-actin. (**b**) Similarly to (**a**), two cytoplasmic proteins, Oho-31 (green) and tight junction membrane protein Arm (red) were found secreted into the lumen; nuclei are stained for DNA with Hoechst 33258 (blue). (**c**) Tumor suppressor protein p127, the product of *l(2)gl* gene (green), and the nucleolar component fibrillarin (red) are found secreted in the lumen; nuclei are stained for DNA with Hoechst 33258 (blue). Fluorescently-tagged constructs (most using GFP-), showed that many fusion proteins were secreted into the lumen. These are exemplified by GFP-Rbp1 (**d**). Examples of proteins monitored via *lacZ*-fusion include the transcription factor Ttk (**e**), the dual-specific LAMMER kinase Doa (**f**), the D subunit of the vacuolar H^+^ vATPase Vha36-1 (**g**) and the transcription factor Fkh (**h**).

By using antibodies we were able to detect numerous proteins inside the gland lumen including cytoskeletal proteins (*e.g*. filamentous actin, p127, β-tubulin, non-muscle myosin II heavy chain, α-spectrin, E-cadherin, fasciclin III, crumbs, *etc*.; Figures 2 and 5; Table 1), cytoplasmic/cytosolic proteins (*e.g*. Doa, Rp21, Rp40, E63, importin-α1, Oho-31, Scribbled, mitochondrial pyruvate dehydrogenase; Figures 2 and 5; Table 1), ER- and Golgi proteins (Rp21, Rp40; Figure 2; Table 1), signaling molecules (*e.g*. α-catenin, Wg, Arm, Rab11, Rop, Ras2; Figures 2 and 5; Table 1), and nuclear or chromosomal proteins including transcription factors and chromatin remodeling proteins (*e.g*. nuclear lamin, p53, BR-C, EcR, Usp, Smrter, E74, E75, Kr-h, Rpd3, Sin3A, *etc*.; Figures 2 and 5; Table 1), or nucleolar protein fibrillarin (Table 1).

Utilization of GFP-/RFP-/YFP-fusion constructs and traps was instrumental in identifying variety of proteins released into lumen. These proteins are exemplified by histone 3, Sgs3, clathrin, Atg8, squid, Rbp1, VhaSFD, Pdi, Grasp65, the α-subunit of Na^+^, K^+^-ATPaseα Corail, UDP-glycosyltransferase Luciole, Ser/Thr casein kinase gilgamesh, zw3 Ser/Thr kinase shaggy**,** RNA-binding RNA-3'-phosphate cyclase Rtc-1**,** Chaperonin Cpn60 ATPase Cocoon, RCC1-like RNA binding protein, Tropomyosin 1/Prefoldin, Hrb98DE *etc*. (Figure 5; Table 2).

A few proteins were followed by using P-element constructs having a *lacZ* fusion and detected by X-gal staining due to *lacZ*-fusion: tramtrack, vATPase subunit D, Doa, ng-1, Antp, Fkh, labial, en, brk, pum, mod, puc, ec, arm, sra *etc*. (Figure 5; Table 3). In conclusion, all proteins we tested, whether by antibody staining or by detecting their fluorescence protein- or LacZ-fusion, had positive signal in the lumen, and thus were being secreted by an apocrine mechanism.

**Figure 6:**
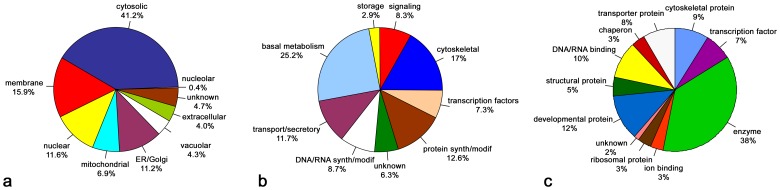
Ontological classification of proteins detected by combination of immunohistochemistry, GFP-/EYFP-/RFP-fusions fluorescence, chromogenic staining of LacZ-insertions and mass spectrometry. The pie shown in (**a**) categorizes proteins according to subcellular localization, while pie (**b**) shows their distribution by biological process, and (**c**) their distribution by cellular/molecular function.

The initial mass spectrometric analysis we performed revealed the presence of 169 proteins in the secretion, the majority of which are cytosolic/cytoplasmic, ER or Golgi-associated components. Altogether with different and independent methods, so far we have identified 292 proteins (for details see Tables 1, 2, 3 and 4). The proteins secreted by this apocrine mechanism include proteins found in many different cellular components: 41.2% are cytosolic proteins, 11.2% are ER chaperones + Golgi proteins, 6.9% are mitochondrial proteins, 15.9% are membrane proteins, and 11.6% are chromosomal, nucleolar and RNA/DNA binding/editing/modifying proteins (Figure 6a). They also reflect a very wide range of biological processes: 11.7% are transport and secretory proteins, 17% are cytoskeletal proteins, 8.3% are involved in signaling, 25.2% are involved in basal metabolism, 7.3% are nuclear proteins and transcription factors, 12.6% are involved in protein synthesis and modification, 2.9% are involved in storage, and 6.3% have unknown functions (Figure 6b). In addition, they also represent many cellular/molecular functions: *e.g*. enzymes 38%, proteins associated with development 12%, DNA and RNA binding proteins 10%, cytoskeletal proteins 9%, transport proteins 8% *etc*. (Figure 6c). From this list is apparent that perhaps all types of cellular proteins are secreted by this apocrine mechanism, and that no specific selection is being made by the cell. However, to validate such a conclusion, we will need to extend this analysis, preferably by MassSpec, to several more hundreds, if not thousands of proteins.

**Table 4:** List of 169 proteins released by apocrine secretion detected by mass spectrometry.

Protein	Accession number	MW (kDa)	Function	Cellular localization
Aconitase	Q9VIE8	85.4	basal metabolism	lipid particle, cytoplasmic
Actin-related protein 87C	P45889	42.7	cytoskeletal	cytoplasmic
Actin 5C	P10987	41.8	cytoskeletal	cytoplasmic
Actin 42A	P02572	41.8	cytoskeletal	cytoplasmic
Actin 57B	P53501	41.8	cytoskeletal	cytoplasmic
Actin 87E	P10981	41.8	cytoskeletal	cytoplasmic
A kinase anchor protein 200	Q9VLL3	79.0	Ras signaling	lipid particle
Alcohol dehydrogenase	P00334	27.0	basal metabolism	cytoplasmic
Aldehyde dehydrogenase	Q9VLC5	57.0	basal metabolism	lipid particle, mitochondrial
Aldolase	P07764	39.0	basal metabolism	cytoplasmic
Annexin X	P22465	35.6	phospholipid binding	cytoplasmic
Apolipophorin	Q9V496	372.7	transport	secreted
Aralar1	Q9VA73	76.7	transport	mitochondrial
Ataxin-2	Q8SWR8	117.5	cytoskeletal	cytoplasmic
Atox1	Q95RR1	7.8	metal ion binding	
Bitesize	Q6XK20	121.5	transport, cytoskeletal	membrane
Black pearl (Mitochondrial import inner membrane translocase subunit Tim16)	Q9VF08	15.7	transport	mitochondrial
BM-40-SPARC	O97365	35.2	calcium binding	extracellular matrix
CathD	Q7K485	42.5	protease	cytoplasmic
Cbl	O46034	52.0	EGF signaling	cell cortex, nuclear
Cecropin A1	P14954	6.8	defense response	secreted
CG10527	Q9W2M4	31.6	basal metabolism	cytoplasmic
CG12140	Q7JWF1	66.0	basal metabolism	cytoplasmic
CG12236	Q9W458	60.8	DNA-binding	nuclear
CG 13993	Q9VMH8	14.7	co-chaperone	endoplasmic reticulum
CG15093 (Probable 3-hydroxyisobutyrate dehydrogenase)	Q9V8M5	33.9	metabolism	mitochondrial
CG1516 (Pyruvate carboxylase)	Q7KN97	130.8	metabolism	lipid particle
CG1523-PA	Q9VAT2	69.6	scaffold	cytoplasmic
CG1640 (Pyridoxal phosphate-dependent aminotransferase)	Q9VYD9	64.0	basal metabolism	cytoplasmic
CG16799	A1ZBX6	21.0	protein modification	cytoplasmic
CG17734	Q8INK7	10.3	signal transduction	membrane(transmembral)
CG30491	Q7JUS1	37.1	metabolism	cytoplasmic
CG32762	Q8IRR6	22.9	unknown	unknown
CG3321	O77134	9.0	H^+^ ATPase	mitochondrial
CG33998	Q6IG52	13.5	unknown	unknown
CG3523	Q9VQL7	266.4	metabolism	lipid particle
CG4151	Q9W4B7	20.5	unknown	unknown
CG4645	Q9VYI1	37.8	transport	membrane
CG5254	Q9V3T2	33.6	transport	membrane
CG5261(putative 2-oxoacid dehydrogenase dihydrolipoyllysine acetyltransferase)	Q7KTK9	54.3	enzyme, metabolism	cytoplasmic
CG5335	Q95SA3	36.7	glycogen metabolsim	cytoplasmic
CG5384	Q9VKZ8	53.7	protease	microtubule associated complex
CG8460	Q9VLS0	45.9	chitinase	secreted
CG8963	Q7K581	63.2	DNA/RNA binding	nuclear
Chickadee (Profilin)	P25843	13.7	cytoskeletal	cytoplasmic, cortical
Chitinase-like protein Idgf4 (Imaginal disk growth factor 4)	Q9W303	48.6	growth factor	secreted
Clathrin heavy chain	P29742	191.2	traffic	vesicle membrane, endosomal
Corazonin receptor	Q9VTW7	64.1	signaling	membrane
C-terminal Src kinase	Q9VGK8	87.2	protein modification	cytoplasmic
dIAP1	Q24306	48.0	apoptosis	cytoplasmic
Dihydropterin deaminase	Q9VMY9	48.9	guanine/pigment metabolism	cytoplasmic
Dispatched	Q9VNJ5	139.0	smo signaling	membrane
drICE	O01382	37.4	apoptosis caspase	cytoplasmic
Egalitarian	Q9W1K4	125.0	RNA transport	nuclear
Elongation factor 1-alpha 1(EF-1-alpha 1)	P08736	50.3	protein synthesis	cytoplasmic, endoplasmic reticulum
Elongation factor 2, isoform A	P13060-1	94.5	protein synthesis	cytoplasmic, endoplasmic reticulum
Elongation factor 2, isoform C	P13060-3	93.1	protein synthesis	cytoplasmic, endoplasmic reticulum
Enolase	P15007	54.3	metabolism	cytoplasmic
Escargot	P25932	52.0	transcription	nuclear
eukaryotic translation initiation factor 4G2	Q9VCH1	266.6	protein synthesis	cytoplasmic, endoplasmic reticulum
Falafel	Q9VFS5	109.3	phosphatase	nuclear, cytoplasmic
Fat body protein 1	Q04691	119.7	transport	extracellular
Ferredoxin	P37193	19.7	transport	mitochondrial
Ferritin 1 heavy chain homologue	Q7KRU8	23.1	transport	extracellular
Ferritin 2 light chain homologue	Q9VA83	25.2	transport	extracellular
FGGY glycerol kinase	Q9W095	64.4	enzyme, metabolism	cytoplasmic
Frizzled 2	Q9VVX3	75.5	Wg/Wnt signaling	cell membrane
Fructose-bisphosphate aldolase 4 alpha	P07764-2	39.6	basal metabolism	cytoplasmic
General odorant-binding protein 99b	Q9VAI6	17.2	signaling	secreted
Gip-like	P36951	29.1	enzyme, metabolism	cytoplasmic
Glyceraldehyde 3 phosphate dehydrogenase 1	P07486	35.4	metabolism	cytoplasmic
Glyceraldehyde 3 phosphate dehydrogenase 2	P07487	35.4	metabolism	cytoplasmic
Glutamate oxaloacetate transaminase 1	Q7K221	46.1	metabolism	cytoplasmic
Glutamate oxaloacetate transaminase 2	Q8IPY3	48.2	metabolism	lipid particle, mitochondrial
Glutathione S transferase D1	P20432	23.9	defense response enzyme	cytoplasmic
Glutathione S-transferase E7	A1ZB72	25.5	defense response enzyme	cytoplasmic
Glutathione S-transferase O3	Q9VSL2	27.7	defense response enzyme	cytoplasmic
GTP-binding nuclear protein Ran (GTPase Ran)	P38545	24.9	transport	nuclear
Heat shock protein cognate 72 (GRP 78)	P29844	72.3	chaperone	endoplasmic reticulum
Heat shock protein 83 (HSP 82)	P02828	81.9	chaperone	cytoplasmic
Heat shock 70 kDa protein cognate 3	P29844	72.3	chaperone	endoplasmic reticulum
Heat shock 70 kDa protein cognate 4	P11147	71.1	chaperone	cytoplasmic, nuclear
Helix loop helix protein 106	Q9VW3	130.0	transcription	nuclear membrane,ER membrane
Hel25E	Q27268	48.7	RNA splicing	nuclear
Heparan sulfate 2-O-sulfotransferase	P25722	41.3	enzyme	Golgi, membrane
Hexokinase A	Q9W330	59.2	metabolism	cytoplasmic
Histone acetyltransferase Tip60	Q960X4	61.2	transcription	nuclear
Hsp70/Hsp90 organizing protein	Q9VPN5	55.7	co-chaperone	cytoplasmic, endoplasmic reticulum
Hsc70Cb	Q9VUC1	88.5	co-chaperone	cytoplasmic, endoplasmic reticulum
IGF-II mRNA-binding protein	Q8IR99	62.7	RNA splicing	nuclear
Inflated (Integrin alpha-PS2)	P12080	140.0	cell adhesion	membrane
Isocitrate dehydrogenase	Q7KUB0	46.6	metabolism	mitochondrial
iso Glutaminyl cyclase	Q7KTY3	40.3	metabolism	mitochondrial
Kenny	Q9GYV5	43.9	immunity	cytoplasmic, nuclear
Kinesin-73	A1ZA18	215.0	cytoskeletal	cytoplasmic
Larval serum protein 2	Q24388	79.0	transport	secreted
Lethal(1)G0255 (fumarate hydratase)	Q8IRQ5	50.5	metabolism	mitochondrial
LSP1 beta	P11996	95.9	storage/transport	secreted
LSP1 gamma	P11997	79.0	storage/transport	secreted
Malic enzyme	Q9VG31	84.6	metabolism	cytoplasmic
Malic enzyme b	Q9VB69	68.6	metabolism	cytoplasmic
MAP kinase kinase 4	O61444	47.5	signaling	cytoplasmic
Minibrain	P49657	65.9	protein modification	nuclear
Molecule interacting with CasL	Q86BA1	525.0	cytoskeleton enzyme	cytoplasmic
Myosin II	Q99323	227.0	cytoskeletal	cytoplasmic, cortical
NADH:ubiquinone reductase 23kD subunit precursor	Q9VF27	24.6	metabolism	membrane
NAT1	Q0E996	104.5	DNA/RNA binding	nuclear, cytoplasmic
NTF2-related export protein 1	Q9V3H8	15.2	transport	nuclear
Nucleoplasmin	Q27415	16.9	chromatin regulator	nuclear
Paramyosin	P35416	74.3	cytoskeletal	cytoplasmic
Pastrel	Q8IQ80	77.4	transport	cytoplasmic
PDGF- and VEGF-related factor 2	Q9VM43	46.9	signaling	membrane
Peptidoglycan recognition protein LC	Q9GNK5	56.1	immunity	membrane
Peptidoglycan-recognition protein-SB2	Q9VV96	20.5	immunity	secreted
Pheromone-binding protein-related protein 3 (Odorant-binding protein 83a)	P54193	17.3	signaling	secreted
Phosphodiesterase 1c	Q9VKE9	67.7	enzyme	cytoplasmic
Phosphofructokinase	P52034	86.6	metabolism	cytoplasmic
Phosphoglucose isomerase	P52029	62.3	metabolism	cytoplasmic
Phosphoglycerate kinase	Q01604	44.0	metabolism	cytoplasmic
Phosphoglyceromutase	Q9VAN7	28.6	metabolism	cytoplasmic
Pi3K92E	P91634	127.0	enzyme	cytoplasmic
Polypeptide N-acetylgalactosaminyltransferase 35A	Q8MVS5	71.8	protein modification	Golgi, membrane
Dnz1 (palmitoyltransferase ZDHHC11)	Q9XTL3	31.7	protein palmitoylation	ER
Phenoloxidase subunit A3	Q9V521	79.3	tanning enzyme, defense response	secreted
Prophenol oxidase A1	Q7K2W6	79.1	tanning enzyme, defense response	secreted
Prophenol oxidase 45	Q9V521	79.3	enzyme	secreted
Pyruvate dehydrogenase kinase	P91622	46.6	metabolism	mitochondrial
Pyruvate kinase	O62619	57.4	metabolism	cytoplasmic
Rac1 (RacA)	P40792	21.4	signaling	membrane
Ran GTPase activating protein	Q9VIW3	66.0	signaling	cytoplasmic
Refractory to sigma P	P14199	65.3	protein tyrosine phosphatase	nuclear
Regucalcin	Q9VYR1	33.6	co-chaperone	cytoplasmic
Rho-kinase	Q9VXE3	160.3	cytoskeletal enzyme	cell cortex
Rho-1	P48148	21.7	signaling	membrane
Ribosomal protein L4	P09180	45.0	translation	ribosome
Ribosomal protein L7-like	Q9VKC1	29.2	translation	ribosome
Ribosomal protein L10	O61231	25.5	translation	ribosome
Ribosomal protein L14	P55841	19.2	translation	ribosome
Ribosomal protein L32	P04359	16.0	translation	ribosome
Rpn5 (Regulatory particle non-ATPase 5)	Q9V3Z4	57.7	proteolysis	proteasome regulatory particle
S-adenosylmethionine decarboxylase	P91931	39.8	enzyme	cytoplasmic
Salivary glue protein Sgs-3	P02840	32.2	extracellular glue	secreted
Scaffold attachment factor B	Q7K1P7	44.4	mRNA splicing	nuclear
Scheggia	Q7KSQ0	34.1	transport	membrane
Serpin 77Ba	Q0E8C8	50.2	defense response	secreted, (extracellular matrix)
Small ribonucleoprotein particle protein SmD3	O44437	15.6	RNA processing	nuclear
Snx6	Q9VLQ9	50.1	vesicular transport	cytoplasmic
α-Spectrin	P13395	280.0	cytoskeletal	cytoplasmic, membrane
Src oncogene at 42A (Tyrosine-protein kinase Src42A)	Q9V9J3	59.1	signaling enzyme	cytoplasmic
Stromal interaction molecule	P83094	64.8	transport	membrane
Supernumerary limbs	Q9VDE3	59.0	proteosomal degradation	cytoplasmic
Synaptojanin	Q5U0V7	134.6	enzyme	
Syndecan	P49415	42.1	signaling	membrane
Tetraspanin 42Ef	Q7K010	24.7	scaffolding/anchoring	membrane
Thioredoxin	Q9W022	15.9	enzyme	cytoplasmic
Thioredoxin reductase-1	P91938	64.3	metabolism	mitochondrial
Transferrin 2	Q9VTZ5	92.3	transport	extracellular
Trehalase	Q9W2M2	67.7	metabolism	cytoplasmic
Triose phosphate isomerase	P29613	26.6	metabolism	cytoplasmic
Tropomodulin	O46231	41.4	cytoskeletal	cytoplasmic
Tropomyosin 1	P06754	39.3	cytoskeletal	cytoplasmic
α-Tubulin84B	P06603	49.9	cytoskeletal	cytoplasmic
α-Tubulin85E (Tubulin alpha-2 chain)	P06604	50.0	cytoskeletal	cytoplasmic
α-Tubulin84D (Tubulin alpha-3 chain)	P06605	49.9	cytoskeletal	cytoplasmic
Vacuolar H^+^ ATPase G subunit	Q9XZH6	13.6	endosomal acidification	endosomes
Vacuolar H^+^-ATPase B subunit	P31409	55.0	endosomal acidification	endosomes
Yorkie	Q45VV3	46.2	transcription	cytoplasmic, nuclear
6-phosphogluconate dehydrogenase	P41572	52.4	metabolism	cytoplasmic
40S ribosomal protein S21	O76927	9.2	translation	cytoplasmic

The molecular weight (kDa) of each protein is listed along with its accession number (SwissProt, UniProt, PIR or TrEMBL) as well as its molecular function and cellular localization.

### Proteins secreted by apocrine mechanism are released sequentially and stay intact (undegraded)

**Figure 7:**
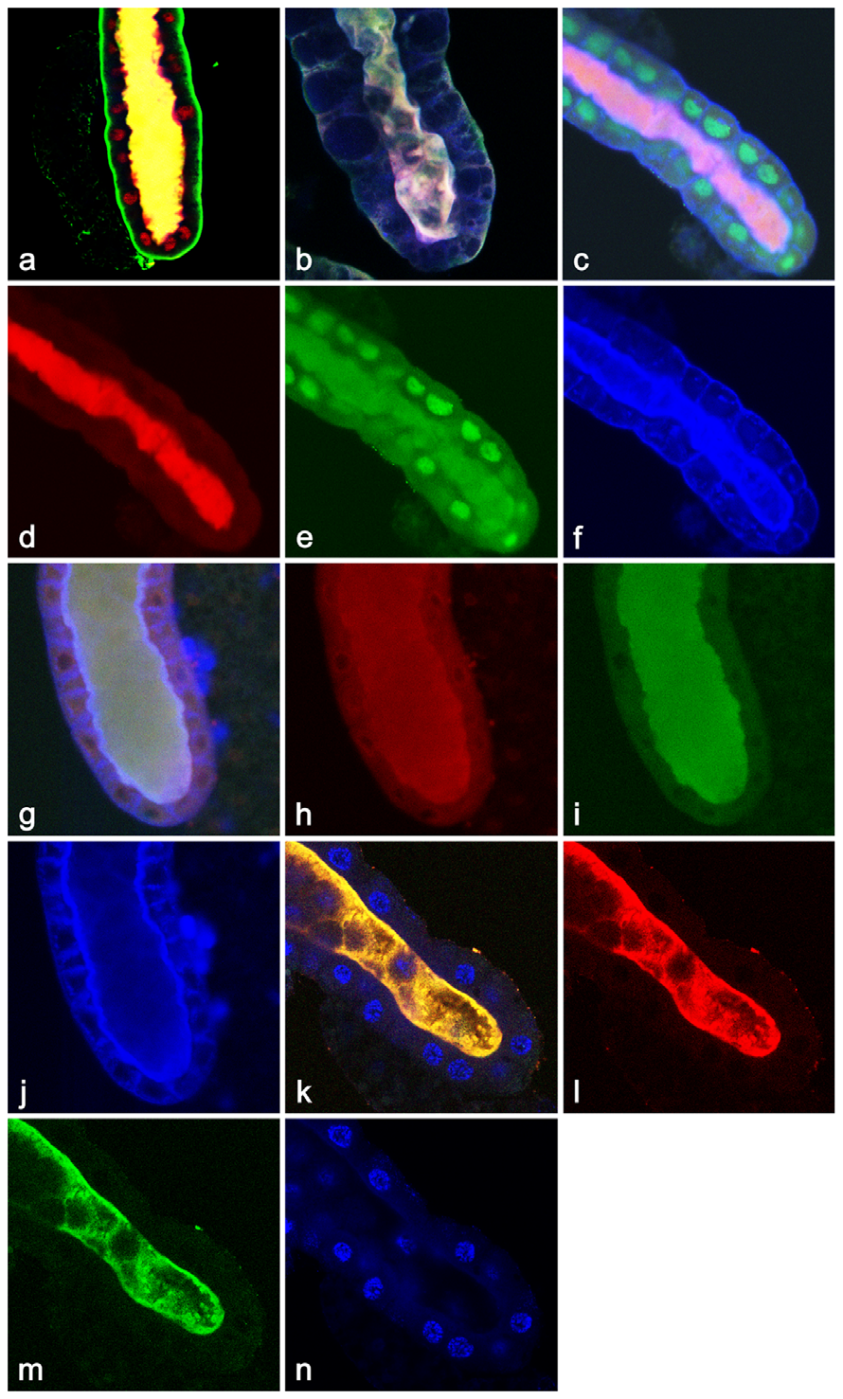
Evidence for the graded temporal release of different proteins by apocrine secretion. (**a**) = At+8.5 hr APF, the ribosomal protein Rp40 (blue) is completely released into lumen, the cortical membrane component α-spectrin (green) was removed from the lateral and apical surfaces but remained at the basal surface, and the nuclear receptor Usp (red) is about half-released into the lumen. (**b**) At +9 hr APF, both the ribosomal protein Rp21 (green) as well as the ecdysone-inducible Ets-like E74 transcription factor (red) are present only in the lumen, whereas there remains significant F-actin (blue) signal on the cortical membranes. (**c**) At the same time (+9 hr APF), the ecdysone-regulated transcription factor and nuclear tumor suppressor are secreted differently: while Kr-h (red (**d**)) is completely extruded into the lumen, p53 (green (**e**)) only starts to be released and the majority of its signal is still detected in nuclei. Although filamentous actin (blue (**f**)) already is being secreted into the lumen, there is detectable signal still visible on cell membranes. (**g**) During +9 to +10 hr APF, the ecdysone-regulated transcription factor BR-C (green (**h**)) is completely released into the lumen, whereas lamin C (red), a component of the nuclear envelope, is only partially released and can be still detected on the nuclear membrane (**i**). Although filamentous actin (blue) is already within the lumen, significant amounts of it still line the cortical cytoskeleton, mainly at the apical membrane (**j**). (**k**) At the end of +10 hr APF both, Rab11 (green (**l**)), a member of the GTPase family of membrane proteins as well as the tumor suppressor transcription factor p53 (red (**m**)) have been completely secreted into the lumen. Hoechst 33258 was used to detect nuclear DNA (blue (**n**)) which stays in nuclei. All confocal images 400×.

The data above suggested that not all proteins are released simultaneously, and that their release might display differential dynamics. In order to scrutinize this possibility, we screened 8–10 hr old prepupal glands, timed at 30 min intervals, with a variety of combinations of antibodies to monitor protein release into the lumen. Figure 7a documents that, for example, at +8.5 hr APF, the ribosomal protein Rp40 (blue) is completely released in lumen, the cortical membrane component α-spectrin (green) becomes removed from the lateral and apical surfaces but remains solely on the basal membrane, while about half of the total nuclear receptor (transcription factor) Usp (red) is released. Interestingly, just about 30 min later, both the ribosomal protein Rp21 (green) and the ecdysone-inducible ets-like E74 transcription factor (red) are present only in the lumen, whereas a significant portion of the F-actin (blue) signal still remains on cortical membranes (Figure 7b). As shown in Figure 7c-f, about at the same time (+9 hr) the ecdysone-regulated transcription factor and the tumor suppressor are secreted differently: while Kr-h (red (d)) is completely extruded into lumen by this time, the p53 (green (e)) has only started to be released and the majority of its signal can be still detected in nuclei. Although filamentous actin (blue (f)) is being already secreted in the lumen, a detectable portion of its signal is still visible on cortical cell membranes. Between +9 and +10 hr of prepupal development, the ecdysone-regulated transcription factor BR-C (green (g, h)) is completely released into the lumen, whereas lamin C (red), a component of the nuclear envelope, is only partially released and can be still detected on the nuclear membrane (g, i). Although filamentous actin (blue) is being already inside the lumen, significant amounts of this protein are still lining the cortical cytoskeleton and mainly apical membrane (Figure 7g, j). By the end of secretory phase (+10 hr APF) both Rab11, a member of the GTPase family of membrane proteins (green (k, l)) as well as p53, the tumor suppressor transcription factor (red (k, m)), similar to the majority of the screened proteins, are completely secreted into the lumen. Hoechst 33258 staining used to detect nuclear DNA (blue (k, n)), was always found only in nuclei.

**Figure 8:**
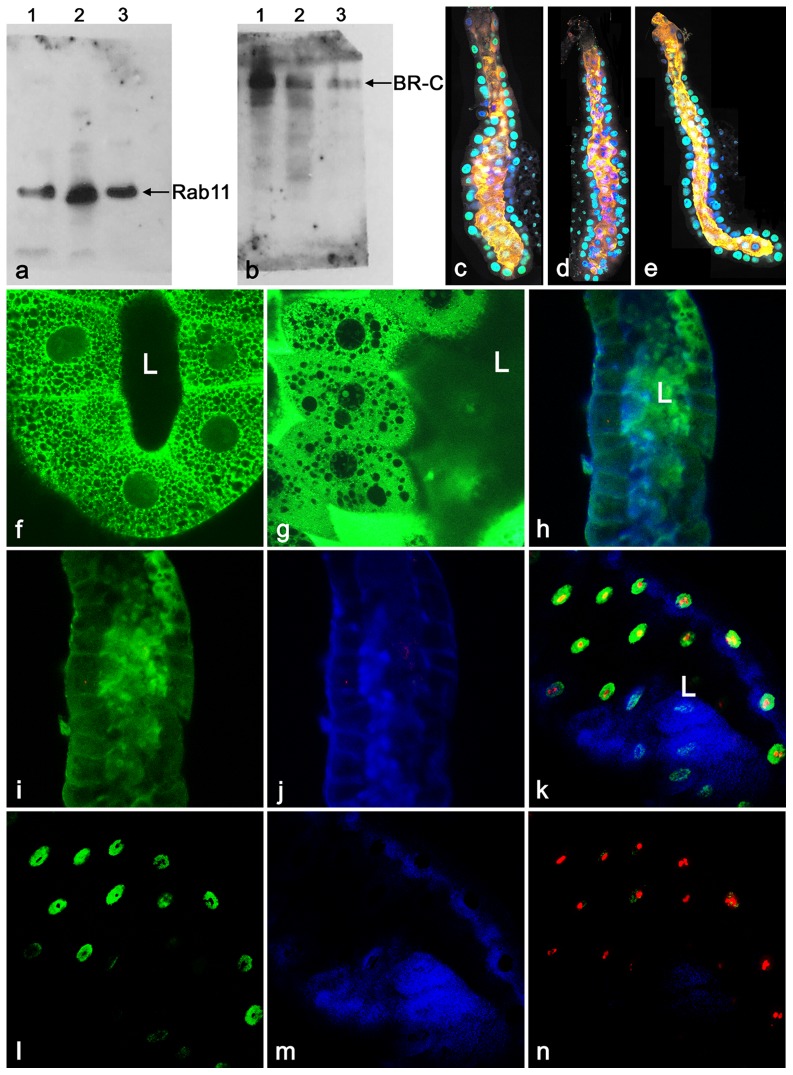
Evidence for apocrine secretion of undegraded proteins and the presence of intact genomic DNA in nuclei, and for the release of mitochondria into lumen. Panels a and b show western blots of secreted proteins isolated from the lumen. (**a**) Rab11 protein was detected in total protein extracts from late larval salivary glands (lane 1), +7 hr APF prepupal salivary glands (lane 2), and the isolated luminal secretion (lane 3). (**b**) The transcription factor BR-C Z1 was detected in total protein extracts from late larval salivary glands (lane 1), +7 hr APF prepupal salivary glands (lane 2), and the isolated luminal secretion from +9–10 hr APF (lane 3). (**c**) In +8–8.5 hr APF prepupae, ribosomal protein Rp40 (green) and β-tubulin (red) are detectable in the lumen of the salivary glands, while the signal for DNA remains nuclear. (**d**) In +9 hr APF prepupae, the ribosomal protein Rp21 (green) and transcription factor E74 (red) are detected in the lumen, while the signal for DNA remains nuclear. (**e**) In +10 hr APF prepupae, both the ribosomal protein p127 (green) and the transcription factor BR-C (red) are detected in the lumen, while the signal for DNA remains nuclear throughout the entire salivary gland, including its columnar, transitional and corpuscular cells; confocal images 80×. (**f**, **g**) Mitochondria are released by apocrine secretion into the lumen as evidenced by chasing a vital Rhodamine 123 signal. In larval as well as early prepupal salivary glands, intact living mitochondria are visible only inside cells (**f**), whereas in +8–10 hr APF prepupae they also can be detected inside the lumen (**g**); both confocal images 630×. This is also consistent with detection of more than dozen of various mitochondrial proteins listed in Tables 1 through 4. In addition, *in situ* hybridization with a mitochondrial genome-specific DNA probe (3'-OH end of mt cytochrome c oxidase I, entire coding sequence of mt tRNA-Leu, and 5'-OH end of mt cytochrome c oxidase II) confirmed the presence of mitochondrial DNA in the secretory material in +9 hr APF prepupae (**h**, **i**, (green)) along with F-actin (**h**, **j**, (blue)). Although nuclear proteins are released by an apocrine mechanism into the lumen, nuclear DNA was never detected in the secretion. When *in situ* hybridization was performed in +9 hr APF prepupae with a probe for a nuclear gene *Doa* locus, signal was found only in nuclei (**k**, **n**, (red)) together with Hoechst 33258 staining DNA (**k**, **l**, (green)), while F-actin was detectable in the lumen (**k**, **m**, (blue)). Remaining confocal images 400×. L in (**f**), (**g**), (**h**) and (**k**)  =  lumen.

As mentioned above, the apocrine secretion in prepupal salivary glands takes place just a few hours prior to programmed cell death (PCD). Therefore, we asked whether the material released from the cells 4 to 6 hr prior to histolysis was already degraded, which would link apocrine secretion with the temporally close senescent fate. We addressed this by isolating secretory material from 8 to 10 hr old prepupal salivary glands, extracting proteins, and probing western blotting with selected antibodies from our collection. As illustrated in the Figure 8a,b, the tested antigens (Rab11 membrane component, BR-C transcription factor) remained intact and were undegraded in the prepupal secretion when these secretions were compared to the total protein extracted from late larval salivary glands. The same results were obtained when extracted secretions were probed on western blot with antibodies against tumor suppressor protein p127, myosin II, Rop, β-tubulin, EcR, Scrib, and Arm (not shown).

As was shown in Figure 7k-n, only proteins, and not nuclear DNA, appear to be released during apocrine secretion. To verify this result for cells of the entire gland, which is composed of columnar, transitional and corpuscular cells, we detected DNA with Hoechst 33258 and various proteins with antibodies at 8, 9 and 10 hr after pupariation. Figure 8c, d and e shows that during all three time points when various proteins are unambiguously secreted, nuclear DNA remains intact in all cells of the gland. Nevertheless, when 8–10 hr old salivary glands are overstained with Hoechst 33258, a very faint DNA signal is detected in the lumen; this was not observed in earlier or later stages of the glands. We speculated that this might be due to the extrusion of whole mitochondria as a part of the secreted material, which was described above. Therefore, we followed mitochondria dynamics using the vital mitochondrial membrane-specific laser dye, Rhodamine 123, uploaded for 10min in living salivary glands. As illustrated in Figure 8f, no Rhodamine-positive signal can be detected in salivary glands prior to secretion, whereas in 9–10 hr old glands, visible mitochondrial fluorescence was found during secretion in the lumen (Figure 8g). To follow this process at the DNA level, we performed *in situ* hybridization with a probe specific to mtDNA. In 10 hr old prepupal salivary glands we were able to detect declining cellular and clear lumenal signal from a digoxygenin/FITC-labeled probe covering three mitochondrial genes in a unique arrangement (3'-OH end of mt cytochrome c oxidase I, entire coding sequence of mt tRNA-Leu, and 5'-OH end of mt cytochrome c oxidase II) (Figure 8h–j). To verify the status of nuclear DNA, a cDNA probe for the single-copy chromosomal gene *Doa*, which encodes a dual-specific LAMMER protein kinase, was hybridized *in situ* to 10-hr old prepupal salivary glands. As illustrated in Figure 8(k–n) , the cDNA probe hybridized crisply only to a single locus within nuclei (red (Figure 8n)) and no extranuclear signal was detected, while F-actin (blue (Figure 8m)) was observed to be released into lumen.

### Vital synthetic activities are retained following apocrine secretion

**Figure 9:**
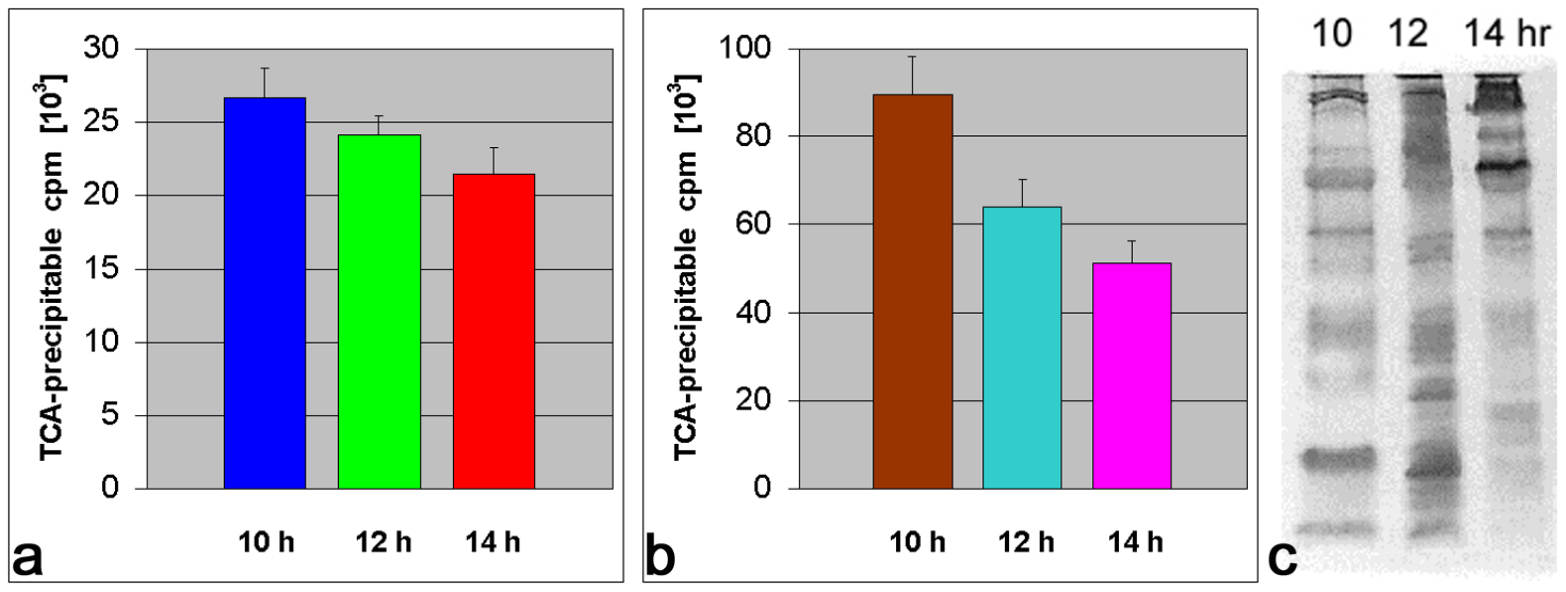
Following apocrine secretion, cells remain transcriptionally and translationally active. Pulse-chase incorporation of [^3^H]-uridine into total RNA in 10, 12 and 14 hr old prepupal salivary glands (**a**) and incorporation of [^35^S]-methionine into proteins detected as TCA-precipitable radioactivity from SDS-protein extracts of 10, 12 and 14 hr old prepupal salivary glands (**b**) show that the cells of the *Drosophila* salivary glands remain viable even after the extrusion of substantial proteinaceous material. The decreasing incorporation rates in prepupae ageing from 10 to 14 hr is likely to reflect a reduction in the available components of the RNA and protein synthesis machinery. However, the salivary glands remain synthetically active and progress along a specific developmental program even following the period of massive protein extrusion: when the protein extracts are resolved by SDS-PAGE and detected using fluorography (**c**) substantially different, but identical when replicated, protein profiles are produced at discrete stages from +10 to +14hours APF.

As protein extrusion takes place a just few hours prior to the execution of programmed cell death, we asked whether salivary gland cells that are losing the majority of their cellular protein components are able to retain basic vital functions. As illustrated in Figure 9a and b, glands in the final phases of protein extrusion (+10 hr APF), as well as glands several hours older (12–14 hr APF) still incorporate radioactively labeled uridine ([^14^C]-uridine or [^3^H]-uridine) and amino acids ([^35^S]-methionine or [^3^H]-leucine) into newly synthesized RNA and proteins, respectively. Furthermore, the pattern of proteins synthesized is not static, but changes as the glands age further (Figure 9c). These prepupal salivary glands also have viable cells as assessed by a dye exclusion test with trypan blue (not shown). Thus, even at time points past the massive, non-canonical apocrine secretion, these glands have cells that are fully alive and continue to maintain a pattern of transcriptional and protein synthetic activities. Indeed, this fits precisely with our understanding of the well-defined puffing pattern of salivary gland polytene chromosomes during this developmental period [78]–[82]. Therefore, this secretory cycle appears to be one of the vital and programmed functions of salivary gland prepupal development and appears to not be associated with PCD.

## Discussion

Apocrine secretion, when compared to well-defined exocytosis, certainly is not a prevalent type of secretory pathway. So far, it has been observed in a limited number of organs or tissues, and studied only in few selected experimental species. In addition, along with holocrine secretion, it is observed only in multicellular metazoan eukaryotes, not microbial eukaryotes such as yeasts that, together with mammalian cell lines, served as the major model organisms to elucidate the molecular determinants of the exocytotic pathway.

Apocrine secretion has been described for mammary glands, Harderian glands of some mammals and birds, the prostate and sweat glands of humans, among other glands [83]. Despite the accumulation of a vast amount of data there remains still some confusion on an unambiguous definition of the apocrine process *per se*. Some authors use apocrine secretion to describe the expulsion of lipids or simple organic materials, whereas proteins are released by exocytosis (*e.g*. milk) [26], [84], [85]. Part of the problem associated with this view of lipid apocrine secretion is the failure to support such claims by clear-cut evidence that would exclude the secretion of proteins. In addition, this view is in striking contrast to the original description and definition of apocrine secretion [38]–[40], [86]–[91] that entails loss of part of cytoplasm accompanied by the presence of apical protrusions and the cytoplasmic fragments in the lumen. Though an oily secretion may not necessarily require an apocrine mechanism to release small droplets, if complex structures such as cytoplasmic fragments are secreted into a lumen, they will hardly be devoid of protein. Our data from *Drosophila* strongly indicate that a heterogenous variety of proteins are the major component of apocrine secretion in the salivary gland. Furthermore, there is abundant evidence from individually studied proteins *e.g*. carbonic anhydrase II from the rat coagulating gland [92], [93], transglutaminase from the prostate [94], [95], an unknown signal peptide lacking protein from the mouse vas deferens (MVDP) [96] that proteins can be released by apocrine mechanism. The reason why specialized individual proteins could appear to be released by apocrine secretion instead of exocytosis is unclear, but one possibility is that they are not individually released at all: the above referenced studies may not have had the tools to examine other components of the secretion and thus their studies were concentrated on a single protein.

As it was eloquently stated by Gesase and Satoh [26] in their review, “The puzzling characteristic of most apocrine glands (meaning mammalian) is that they also secrete via exocytosis [84], [97]–[107]. In some glands exocytosis is predominant while in others apocrine secretion become the major pathway for secretion. In some glands apocrine secretion occurs at a low level as compared to exocytosis [108], [109], and in most cases it does not allow detailed morphological observations.“ To this end, the authors neither provide evidence nor discuss whether apocrine secretion and exocytosis take place at the same time or are separate processes. In addition, these conclusions were made solely by studying mammalian apocrine systems. The *Drosophila* salivary glands are famously known for their synthesis and subsequent massive exocytosis of secretory Sgs glycoproteins that serve as a glue to cement the newly forming puparium to a substrate [110], [111]. Expression of the *Sgs* genes, and synthesis of Sgs proteins occurs during the last 16–20 hours of *Drosophila* larval life [112]–[115]. Secretory granules are released during a two hr period by exocytosis taking place about four hr after a pulse of ecdysone triggers the initiation of metamorphosis. The expectoration of the exocytosed glue from lumen takes place some four hr later during the pupariation of the immobile larva [52], [111], [116], [117]. It is only 8 to 10 hr later that the same salivary glands display apocrine secretion of the very complex proteinaceous mixture, we describe here. Thus, typical exocytosis is separated from the later apocrine secretion in the *Drosophila* salivary glands by a 14 to 16 hr period. Although it may appear as a relatively short time in a mammalian world, it is a period of rapid and dramatic change in this insect. In response to metamorphic pulse of the steroid hormone ecdysone, the relatively mobile and actively feeding larva stops feeding, enters a short wandering stage, become motionless, pupariates and then enters an early pupal stage. The larva undergoes dramatic morphogenetic changes that are associated with numerous and complex biochemical and cellular events. Therefore, the 14 to 16 hr period between exocytosis and apocrine secretion can be considered as a substantial time interval and it is significant that these two apparently separate and independent processes are exercised by the very same cells. To answer the question of whether these two processes are truly separate and independent, the immense potential of *Drosophila* model system can be used for molecular genetic dissection of exocytosis from apocrine secretion.

Finding that some proteins in *Drosophila* salivary glands are released by apocrine secretion earlier and other proteins later documents that this is highly regulated process. This also opens up a potentially new area for further research. We cannot unambiguously infer what categorical features of proteins determine earlier versus later release. For example, the order of release does not appear to be based on nuclear versus cytoplasmic localization: some nuclear proteins such as Smrter corepressor are released prior to the cytoplasmic homologue of Sec-1, Rop (see Figures 2 and 7). Moreover, cytoskeletal protein F actin was released at least in 2 phases, even when several other categories of unrelated proteins are secreted. From an ultrastructural perspective, the early phases of secretion can seem to have more soluble proteins extruded, whereas larger pieces of cytoplasm, which are harder to solubilize, are released at later stages. However, we have seen at low frequency larger pieces of the cytoplasm even in very early phases. A consideration in reflecting on these data is that it is easier to detect the occurrence of such „less soluble“ material at later stages because the released materials are being accumulated in the lumen over a secretory phase that lasts two hours, which increases the chances for the detection of larger pieces. When we investigated the order of protein secretion during this 2 hr time window using antibody staining, we found that it showed highly reproducible regularity. From data collected now we can conclude that α-catenin, EcR or p127 can be used as markers for secretion during the 1^st^ hour, BR-C, Rpd3 and Rop as markers for secretion during the 2^nd^ hour, and p55, Grasp65 or lamin as markers for secretion during the 3^rd^ hour. To shed more light on the molecular mechanism that controls this gradual release of proteins, it will be helpful to identify more secreted proteins in a time-lapse fashion, using both a microscopical as well as mass spectrometric approaches.

Nonetheless, a quite interesting point already can be made. It is widely accepted that the implementation of the secretory and apoptotic fates of the larval and prepupal *Drosophila* salivary glands is under the temporal control of ecdysone and the ecdysone transcriptional cascade [118]–[121]. In this study we detected several crucial components of the ecdysone signaling cascade, notably EcR, Usp, Tai, BR-C, E74, E75, and Kr-h to be released by apocrine secretion in the period of time shortly prior to the small prepupal pulse of ecdysteroids. This raises two questions: Why would such important factors be released just prior to when they will be required once again? Are they not missing when the new pulse of ecdysone arrives? First of all, we expect that minimal amounts of each protein must remain in the salivary gland cells, and second, as shown by incorporation of radioactive [^3^H]-uridine and [^35^S]-methionine into RNA and proteins, *de novo* synthesis of gene products is not stopped even during and after apocrine secretory cycle. Furthermore, our immunohistochemical data show that by the end of secretory cycle, the beginning of the 11^th^ hour of puparial development, some of these as well as other protein components are again detectable at their *in situ* locations. Although previous electron microscopical studies on the relevant prepupal period of *Drosophila* salivary glands [52], [115], [116], [122] failed to identify apocrine secretion, they did demonstrate that even immediately after secretion and prior to PCD, the cells appear healthy and are replete with all previously observed organelles and structures. These results are consistent with our proposal that there is an active and continuous renewal of cellular components. This is another, crucial attribute for apocrine secretion, and a feature that differentiates it from holocrine secretion. It is important to stress that apocrine secretion happens just (3–4 hr) prior to the execution phase of PCD in the salivary glands. Thus, we cannot rule out the possibility that at least some of the cellular components necessary for the fully functional execution of PCD are being synthesized *de novo* during this short interval. On the other hand, the relatively large salivary gland cells' utilization of massive apocrine secretion to rid themselves of „surplus“ protein and other cellular contents may facilitate the upcoming apoptotic process by decreasing the amount of substrates and making senescence more efficient.

When secretion has been completed, cells exhibit a loss of about 80% of their protein constituents, based on a decrease in fluorescence signal. We suspect that this process is the same as one described by Sarmiento and Mitchell [123], called the salivary glands “second secretory cycle“. This non-canonical secretion occurs 4 to 6 hr prior to the period, 14 to 16 hr APF, during which salivary glands undergo PCD [47], [52], [124]–[126]. Though we originally expected that this heavy secretion was directly related to PCD, assessments of cell viability, *e.g*. by trypan blue staining, clearly show that the salivary gland cells remain alive at this time, even maintaining high levels of synthetic activities as reflected in the incorporation of [^14^C]-uridine or [^3^H]-uridine and amino acids ([^35^S]-methionine or [^3^H]-leucine) into newly synthesized RNA and proteins, respectively. Indeed, our results are in good agreement with those of Tissiéres *et al*. [127] and Zhimulev *et al*. [128] who monitored protein synthesis in larval and prepupal SGs in relation to puffing patterns, which are well-documented to continue even after this period [79]–[82], [129], [130], and indicate the continued viability of the glands.

Proteasomal degradation is known to be permanent and continuous process in many if not all cells of the organism [131]–[136]. Thus, one can expect that it occurs also in prepupal salivary glands. If “used” and unwanted proteins are continuously removed by proteasomal degradation, and the removal of such proteins were one goal of apocrine secretion, then some signs of this degradation should also be detectable in protein extracts of isolated salivary gland secretions. However, we were unable to detect any low-molecular weight degradation products, even on overexposed X-ray films from western blots. As we detected undegraded proteins in the released material by western blotting as well as morphologically perfect pieces of cellular structures in the lumen by electron microscopy, this documents that the apocrine secretion process is a real secretory activity with a different functional significance. We conclude that apocrine secretion is selective process because only undegraded proteins are released whereas those targeted for proteasomal degradation are retained in cells. This is a novel and important attribute of apocrine secretion.

Interestingly, many of the proteins identified in our initial top-down proteomic analysis or by microscopy are encoded by genes recovered by Maybeck and Roper [137] in their targeted gain-of-function screen for embryonic salivary gland morphogens. These include genes such as *chic*, *egl*, *btsz*, *Arp87C*, and others, and according to the modENCODE project and FlyAtlas tissue expression data [138], [139], such genes are known to be moderately to highly expressed in salivary glands. This indicates that these genes, which are important for embryonic morphogenesis of this tissue remain active and are highly or increasingly expressed throughout the life of the gland, and so may be essential or vital for maintaining this organ‘s identity, structure or function until the realization of cell death. On the other hand, several polypeptides detected by mass spectrometry, such as transferrin, larval serum proteins (yolk proteins) are almost surely not endogenous products of salivary glands, but exemplary representatives of hemolymph or fat body proteins. This strongly indicates that these are transudated, similar to previous observations *e.g*. for albumin in mammalian tears [140]–[143].

Though our proteomic analysis has clear limitations, it was very instrumental for determining a large variety of different and unrelated proteins that are released by apocrine secretion from the salivary glands. It has supported and extended our initial understanding, gained by antibody screening and tracking labeled proteins, of the size of the constellation of proteins that are secreted. We are currently utilizing both, the MALDI-TOF/TOF and the ESI based nano-HPLC-MS/MS shotgun proteomic methods to better characterize this set of proteins.

When a *lacZ* expressions pattern is assessed, only those constructs which insert *lacZ* inside the coding sequence can be used to trace particular protein. Enhancer traps, for example, can show a functional β-galactosidase staining pattern when *lacZ* is expressed from an exogenous and heterologous reporter. In such cases, when the X-Gal substrate is converted to a blue-colored precipitate, it is also trapped into the transportation machinery for delivery by the apocrine pathway to the secretory lumen. Therefore, we only considered a protein to be secreted if its protein-coding fusion with *lacZ* revealed luminal β-galactosidase staining. Although the majority of *lacZ* constructs showing luminal staining were enhancer traps, their potential inclusion would not significantly change the distribution of proteins shown in Figure 6. However, this finding has another and more important implication: it shows that even heterologous proteins without an evident internal function are trapped into the recruiting and transportation system used by apocrine secretory machinery. This differs substantially from exocytosis, and also offers a novel opportunity to trace the recruitment and transportation phases of the apocrine process by using foreign heterologous tools. One hypothesis, testable using the genetic tools available in *Drosophila*, is that the trapping of β-galactosidase into the apocrine secretion machinery indicates that this system is not specific and can recruit all available proteins. Compared to endogenous cellular proteins, free bacterial β-galactosidase has no obvious function in the *Drosophila* salivary glands. Before it can be found in the lumen of late prepupal glands, it is found almost everywhere, and is mostly cytoplasmic. In contrast, all endogenous internal proteins are at their native location (nuclei, mitochondria, ER, Golgi, membrane *etc*.), and have their own targeting sequences. Therefore, we anticipate that to the ability to include all these different and heterogenous proteins into a single secretory pathway requires an extremely powerful and efficient recruitment machinery. It may likely involve a novel and unknown mechanism of posttranslational modification.

Our data, which are without precedent, clearly show that *Drosophila* salivary glands are actively engaged in apocrine secretion, which is distinct from holocrine secretion that is accompanied by the release of nuclei [144]–[146]. Even under the most massive protein secretion by the apocrine pathway, we never detected release of nuclear DNA, even though nuclear and nucleolar proteins were secreted. Thus, this feature can be considered as one of the hallmarks that distinguish apocrine from holocrine secretion. The above mentioned discrepancy between the few proteins found in apocrine secretion in mammals and the nearly entire proteome in the apocrine secretion of *Drosophila* presents a new and compelling challenge. One possibility is that apocrine secretion in mammalian and other animal systems is also utilized to release many more proteins than appreciated so far, and may serve as a good alternative to exocytosis, which is known to be devoted to the frequently repeated secretion of a few, highly specialized products. Our discovery provides a promising opportunity that this and hopefully other challenges associated with such noncanonical secretion can be addressed in the near future. The molecular and genetic tools so specifically available in *Drosophila* will allow us to use this model organism to dissect the components of the apocrine signaling pathway. Lastly, but not least, these findings are likely to have practical ramifications for medicine. Various disorders of apocrine secretion are known to be associated with more than two dozen diseases, including breast, salivary and skin tumors [29], [31]–[35].

## References

[1] Jahn R (2004) Principles of exocytosis and membrane fusion. Ann N Y Acad Sci 1014: 170–178. 15153432 10.1196/annals.1294.018

[2] Rutter GA, Tsuboi T (2004) Kiss and run exocytosis of dense core secretory vesicles. Neuroreport 15: 79–81. 15106835 10.1097/00001756-200401190-00016

[3] Südhof TC (2004) The synaptic vesicle cycle. Annu Rev Neurosci 27: 509–547. 15217342 10.1146/annurev.neuro.26.041002.131412

[4] Chieregatti E, Meldolesi J (2005) Regulated exocytosis: new organelles for non-secretory purposes. Nat Rev Mol Cell Biol 6: 181–187. 15688003 10.1038/nrm1572

[5] Barclay JW, Morgan A, Burgoyne RD (2005) Calcium-dependent regulation of exocytosis. Cell Calcium 38: 343–353. 16099500 10.1016/j.ceca.2005.06.012

[6] Snyder DA, Kelly ML, Woodbury DJ (2006) SNARE complex regulation by phosphorylation.Cell Biochem Biophys. 45: 111–123. 10.1385/CBB:45:1:111 16679567

[7] Westerink RH (2006) Targeting exocytosis: ins and outs of the modulation of quantal dopamine release. CNS Neurol Disord Drug Targets 5: 57–77. 16613554 10.2174/187152706784111597

[8] Leitzell K (2007) Synaptotagmin: is 2 better than 1? J Neurosci 27: 4231–4232. 17442806 10.1523/JNEUROSCI.0668-07.2007 PMC6672327

[9] Deak F, Xu Y, Chang WP, Dulubova I, Khvotchev M, et al (2008) Munc18-1 binding to the neuronal SNARE complex controls synaptic vesicle priming. J Cell Biol 184: 751–764. 10.1083/jcb.200812026 PMC2686405 19255244

[10] Beck R, Ravet M, Wieland FT, Cassel D (2009) The COPI system: Molecular mechanisms and function. FEBS Lett 583: 2701–2709. 19631211 10.1016/j.febslet.2009.07.032

[11] Südhof TC, Rothman JE (2009) Membrane fusion: grappling with SNARE and SM proteins. Science 323: 474–477. 19164740 10.1126/science.1161748 PMC3736821

[12] Anantharam A, Onoa B, Edwards RH, Holz RW, Axelrod D (2010) Localized topological changes of the plasma membrane upon exocytosis visualized by polarized TIRFM. J Cell Biol 188: 415–428. 20142424 10.1083/jcb.200908010 PMC2819686

[13] He B, Guo W (2010) The exocyst complex in polarized exocytosis. Curr Opin Cell Biol 21: 537–542. 10.1016/j.ceb.2009.04.007 PMC2725219 19473826

[14] Blank U (2011) The mechanisms of exocytosis in mast cells. Adv Exp Med Biol 716: 107–122. 21713654 10.1007/978-1-4419-9533-9_7

[15] Keren K (2011) Cell motility: the integrating role of the plasma membrane. Eur Biophys J 40: 1013–1027. 21833780 10.1007/s00249-011-0741-0 PMC3158336

[16] Jahn R, Fasshauer D (2012) Molecular machines governing exocytosis of synaptic vesicles. Nature 490: 201–207. 23060190 10.1038/nature11320 PMC4461657

[17] Porat-Shliom N, Milberg O, Masedunskas A, Weigert R (2013) Multiple roles for the actin cytoskeleton during regulated exocytosis. Cell Mol Life Sci 70: 2099–2121. 22986507 10.1007/s00018-012-1156-5 PMC4052552

[18] Malsam J, Kreye S, Söllner TH (2008) Membrane fusion: SNAREs and regulation. Cell Mol Life Sci 65: 2814–2832. 18726177 10.1007/s00018-008-8352-3 PMC11131752

[19] Rizo J, Rosenmund C (2008) Synaptic vesicle fusion. Nat Struct Mol Biol 15: 665–674. 18618940 10.1038/nsmb.1450 PMC2519048

[20] Saraste J, Dale HA, Bazzocco S, Marie M (2009) Emerging new roles of the pre-Golgi intermediate compartment in biosynthetic-secretory trafficking. FEBS Lett 583: 3804–3810. 19887068 10.1016/j.febslet.2009.10.084

[21] Walter AM, Wiederhold K, Bruns D, Fasshauer D, Sørensen JB (2010) Synaptobrevin N-terminally bound to syntaxin-SNAP-25 defines the primed vesicle state in regulated exocytosis. J Cell Biol 188: 401–413. 20142423 10.1083/jcb.200907018 PMC2819690

[22] Shen J, Tareste DC, Paumet F, Rothman JE, Melia TJ (2007) Selective activation of cognate SNAREpins by Sec1/Munc18 proteins. Cell 128: 183–195. 17218264 10.1016/j.cell.2006.12.016

[23] Maximov A, Tang J, Yang X, Pang ZP, Südhof TC (2009) Complexin controls the force transfer from SNARE complexes to membranes in fusion. Science 323: 516–521. 19164751 10.1126/science.1166505 PMC3235366

[24] Kasai H, Takahashi N, Tokumaru H (2012) Distinct initial SNARE configurations underlying the diversity of exocytosis. Physiol Rev 92: 1915–1964. 23073634 10.1152/physrev.00007.2012

[25] Satoh Y, Gesase AP, Habara Y, Ono K, Kanno T (1996) Lipid secretory mechanisms in the mammalian harderian gland. Microsc Res Tech 34: 104–110. 8722703 10.1002/(SICI)1097-0029(19960601)34:2<104::AID-JEMT2>3.0.CO;2-S

[26] Gesase AP, Satoh Y (2003) Apocrine secretory mechanism: recent findings and unresolved problems. Histol Histopathol 18: 597–608. 12647810 10.14670/HH-18.597

[27] Vegliante F, Hasenfuss I (2012) Morphology and diversity of exocrine glands in lepidopteran larvae. Annu Rev Entomol 57: 187–204. 21910636 10.1146/annurev-ento-120710-100646

[28] Griffith JR (2005) Isolated areolar apocrine chromhidrosis. Pediatrics 115: e239–241. 15629957 10.1542/peds.2004-1561

[29] Khalbuss WE (2005) Cytomorphology of rare malignant tumors of the breast. Clin Lab Med 25: 761–775. 16308090 10.1016/j.cll.2005.08.004

[30] Shah N (2005) Hidradenitis suppurativa: a treatment challenge. Am Fam Physician 72: 1547–1552. 16273821

[31] Crowson AN, Magro CM, Mihm MC (2006) Malignant adnexal neoplasms. Mod Pathol 2: S93–S126. 10.1038/modpathol.3800511 16446719

[32] Krbec AC (2007) Current understanding and management of hidradenitis suppurativa. J Am Acad Nurse Pract 19: 228–234. 17489955 10.1111/j.1745-7599.2007.00219.x

[33] O‘Malley FP, Bane A (2008) An update on apocrine lesions of the breast. Histopathology 52: 3–10. 18171412 10.1111/j.1365-2559.2007.02888.x

[34] Chi AC, Mapes IL, Javed T, Neville BW (2010) Epidermal choristoma of the oral cavity: report of 2 cases of an extremely rare entity. J Oral Maxillofac 68: 451–455. 10.1016/j.joms.2009.04.120 20116722

[35] Elayat G, Selim AG, Wells CA (2010) Cell turnover in apocrine metaplasia and apocrine adenosis of the breast. Ann Diagn Pathol 14: 1–7. 20123450 10.1016/j.anndiagpath.2009.05.001

[36] Gjorevski N, Nelson CM (2011) Integrated morphodynamic signalling of the mammary gland. Nat Rev Mol Cell Biol 12: 581–593. 21829222 10.1038/nrm3168

[37] Tincani A, Andreoli L, Cavazzana I, Doria A, Favero M, et al (2013) Novel aspects of Sjögren's syndrome in 2012. BMC Med 11: 93 doi: 10.1186/1741-7015-11-93 23556533 10.1186/1741-7015-11-93 PMC3616867

[38] Purkinje J (1833) Review of Burdach's Die Physiologie der Erfahrungswissenschaft. Jahrb Wissen Kritik 1: 789–796.

[39] Velpeau A (1839) Aiselle. In: Dictionnaire de Médecine, un Répertoire Général des Sciences Médicales sous la Rapport Théorique et Practique, vol. 2. Paris: Bechet Jeune. pp. 86–109.

[40] Verneuil A (1854) Études sur les tumeurs de la peau; des quelques maladies des glandes sudoripares. Arch Gén Méd 4: 447–468.

[41] Constantinou C, Widom K, Desantis J, Obmann M (2008) Hidradenitis suppurativa complicated by squamous cell carcinoma. Am Surg 74: 1177–1181. 19097532

[42] Lasko LA, Post C, Kathju S (2008) Hidradenitis suppurativa: a disease of apocrine gland physiology. JAAPA 21: 3–25. 10.1097/01720610-200811000-00006 19105543

[43] Sellheyer K, Krahl D (2008) What causes acne inversa (or hidradenitis suppurativa)? - the debate continues. J Cutan Pathol 35: 701–703. 18582247 10.1111/j.1600-0560.2008.01073.x

[44] Grant A, Gonzalez T, Montgomery MO, Cardenas V, Kerdel FA (2010) Infliximab therapy for patients with moderate to severe hidradenitis suppurativa: a randomized, double-blind, placebo-controlled crossover trial. J Am Acad Dermatol 62: 205–217. 20115947 10.1016/j.jaad.2009.06.050

[45] Mozeika E, Jemec GB, Nürnberg BM (2011) Hedgehog pathway does not play a role in hidradenitis suppurativa pathogenesis. Exp Dermatol 20: 841–842. 21824197 10.1111/j.1600-0625.2011.01344.x

[46] Blok JL, van Hattem S, Jonkman MF, Horváth B (2013) Systemic therapy with immunosuppressive agents and retinoids in hidradenitis suppurativa: a systematic review. Br J Dermatol 168: 243–252. 23106519 10.1111/bjd.12104

[47] Farkaš R, Mechler BM (2000) The timing of *Drosophila* salivary gland apoptosis displays an *l(2)gl*-dose response. Cell Death Differ 7: 89–101. 10713724 10.1038/sj.cdd.4400621

[48] Ashburner M, Thompson JN (1978) The laboratory culture of *Drosophila* In: Ashburner M, Wright TRF, editors. The Genetics and Biology of *Drosophila*, vol. 2a. London and New York: Academic Press, pp. 1–109.

[49] Ransom R (1982) Techniques. In: Ransom R. editor. A Handbook of *Drosophila* Development. Amsterdam and New York: Elsevier Biomedical Press. pp. 1–30.

[50] Lindsley DL, Zimm GG (1992) The Genome of *Drosophila melanogaster*. San Diego, New York and Lodon:Academic Press. 1133p.

[51] Farkaš R (1991) Simple method for high efficiency pulse labelling of proteins and nucleic acids in larval salivary glands of *Drosophila* . Dros Inf Serv 70: 244–246.

[52] Farkaš R, Šuťáková G (1998) The ultrastructural changes of larval and prepupal salivary glands of *Drosophila* cultured *in vitro* with ecdysone. In Vitro Cell Devel Biol 34: 813–823. 10.1007/s11626-998-0036-7 9870531

[53] Laemmli UK (1970) Cleavage of structural proteins during the assembly of the head of bacteriophage T4. Nature 227: 680–685. 5432063 10.1038/227680a0

[54] Weber K, Osborn M (1969) The reliability of molecular weight determinations by dodecyl sulfate-polyacrylamide gel electrophoresis. J Biol Chem 244: 4406–4411. 5806584

[55] Oakley BR, Kirsch DR, Morris NR (1980) A simplified ultrasensitive silver stain for detecting proteins in polyacrylamide gels. Anal Biochem 105: 361–363. 6161559 10.1016/0003-2697(80)90470-4

[56] Laskey RA, Mills AD (1975) Quantitative film detection of ^3^H and ^14^C in polyacrylamide gels by fluorography. Eur J Biochem 56: 335–341. 1175627 10.1111/j.1432-1033.1975.tb02238.x

[57] Bellen HJ, O'Kane CJ, Wilson C, Grossniklaus U, Pearson RK, et al (1989) P-element-mediated enhancer detection: a versatile method to study development in *Drosophila* . Genes Dev 3: 1288–1300. 2558050 10.1101/gad.3.9.1288

[58] Kobayashi S, Okada M (1993) A double staining technique using 5-bromo-4-chloro-3- indolyl-β-D-galactopyranoside (X-gal) and immunoperoxidase in whole *Drosophila* embryos. Biotech Histochem 68: 237–239. 8218577 10.3109/10520299309104704

[59] Tautz D, Pfeifle C (1989) A non-radioactive *in situ* hybridization method for the localization of specific RNAs in *Drosophila* embryos reveals translational control of the segmentation gene *hunchback* . Chromosoma 98: 81–85. 2476281 10.1007/BF00291041

[60] Clary DO, Wahleithner JA, Wolstenholme DR (1983) Transfer RNA genes in *Drosophila* mitochondrial DNA: related 5' flanking sequences and comparisons to mammalian mitochondrial tRNA genes. Nucleic Acids Res 11: 2411–2425. 6304652 10.1093/nar/11.8.2411 PMC325893

[61] de Bruijn MH (1983) *Drosophila melanogaster* mitochondrial DNA, a novel organization and genetic code. Nature 304: 234–241. 6408489 10.1038/304234a0

[62] Yun B, Farkaš R, Lee K, Rabinow L (1994) The *Doa* locus encodes a member of a newprotein kinase family and is essential for eye and embryonic development in *Drosophilamelanogaster* . Genes Dev 8: 1160–1173. 7926721 10.1101/gad.8.10.1160

[63] Shevchenko A, Tomas H, Havlis J, Olsen JV, Mann M (2006) In-gel digestion for mass spectrometric characterization of proteins and proteomes. Nature Protoc 1: 2856–2860. 17406544 10.1038/nprot.2006.468

[64] Rappsilber J, Mann M, Ishihama Y (2007) Protocol for micro-purification, enrichment, pre-fractionation and storage of peptides for proteomics using StageTips. Nature Protocols 2: 1896–1906. 17703201 10.1038/nprot.2007.261

[65] Řehulková H, Chalupová J, Šebela M, Řehulka P (2010) A convenient purification and preconcentration of peptides with α-cyano-4-hydroxycinnamic acid matrix crystals in a pipette tip for matrix-assisted laser desorption/ionization mass spectrometry. J Mass Spectrom 45: 104–111. 19927305 10.1002/jms.1698

[66] Kushida H (1964) Improved methods for embedding with Durcupan. J Electron Micros 13: 139–144. 4862357

[67] Kushida H (1966) Further improved method for embedding with Durcupan. J Electron Micros 15: 94–95. 5965757

[68] Glauert AM (1975) Fixation, dehydration and embedding of biological specimens. In: Glauert AM editor. Practical Methods in Electron Microscopy, vol. 3. Amsterdam and New York: North-Holland American Elsevier. pp. 5–186.

[69] Mráz P, Malatín D, Polónyi J (1982) Modifications of embedding tissues in durcupan ACM (Fluka) based on viscosity index measurements. Z Mikrosk Anat Forsch 96: 130–137. 7102032

[70] Watson ML (1958) Staining of tissue sections for electron microscopy with heavy metals. J Biophys Biochem Cytol 4: 475–478. 13563554 10.1083/jcb.4.4.475 PMC2224499

[71] Reynolds ES (1963) The use of lead citrate at high pH as an electron-opaque stain in electron microscopy. J Cell Biol 17: 208–211. 13986422 10.1083/jcb.17.1.208 PMC2106263

[72] Sato T (1968) A modified method for lead staining of thin sections. J Electron Microsc 17: 158–159. 4177281

[73] Mazza A, Tufano CA, Casale A, Felluga B (1981) A simple and reliable stain for routine microscope observation of ultrathin sections. J Submicrosc Cytol 13: 473–478. 6174736

[74] Beňo M, Liszeková D, Farkaš R (2007) Processing of soft pupae and uneclosed pharate adults of *Drosophila* for scanning electron microscopy. Microsc Res Tech 70: 1022–1027. 17661387 10.1002/jemt.20507

[75] Nation JL (1983) A new method using hexamethyldisilazane for preparation of soft insect tissues for scanning electron microscopy. Stain Technol 58: 347–351. 6679126 10.3109/10520298309066811

[76] Kennedy JR, Williams RW, Gray JP (1989) Use of Peldri II (a fluorocarbon solid at room temperature) as an alternative to critical point drying for biological tissues. J Electron Microsc Tech 11: 117–125. 2709130 10.1002/jemt.1060110205

[77] Bray DF, Bagu J, Koegler P (1993) Comparison of hexamethyldisilazane (HMDS), Peldri II, and critical-point drying methods for scanning electron microscopy of biological specimens. Microsc Res Tech 26: 489–495. 8305726 10.1002/jemt.1070260603

[78] Ashburner M (1970) Function and structure of polytene chromosomes during insect development. In: Beament JWL, Treherne JE, Wigglesworth VB, editors. Advances in Insect Physiology vol. 7. New York and London: Academic Press. pp. 1–95.

[79] Ashburner M (1972) Puffing patterns in *Drosophila melanogaster* and related species. In: Beerman W, editor. Developmental Studies on Giant Chromosomes. Berlin, Heidelberg, New York: Springer-Verlag. pp. 101–151. 10.1007/978-3-540-37164-9_5 4198830

[80] Richards GP (1976) The control of prepupal puffing patterns *in vitro*: implications for prepupal ecdysone titres in *Drosophila melanogaster* . Dev Biol 48: 191–195. 812742 10.1016/0012-1606(76)90057-9

[81] Richards GP (1976) Sequential gene activation by ecdysone in polytene chromosomes of *Drosophila melanogaster*. IV. The mid prepupal period. Dev Biol 54: 256–263. 825404 10.1016/0012-1606(76)90303-1

[82] Ashburner M, Berendes HD (1978) Puffing of polytene chromosomes. In: Ashburner M, Wright TRF, editors. The Genetics and Biology of *Drosophila*, vol 2b. London and New York: Academic Press. pp. 315–395.

[83] Kurosumi K, Shibasaki S, Ito T (1984) Cytology of the secretion in mammalian sweat glands. Int Rev Cytol 87: 253–329. 6370891 10.1016/s0074-7696(08)62445-6

[84] Gesase AP, Satoh Y, Ono K (1995) G-protein activation enhances Ca^2+^-dependent lipid secretion of the rat Harderian gland. Anat Embryol 192: 319–328. 8554165 10.1007/BF00710101

[85] Gesase AP (2007) Apocrine secretory processes in the goblet cells of rat colon following stimulation with carbamylcholine. Ital J Anat Embryol 112: 117–129. 17687876

[86] Schiefferdecker P (1922) Die Hautdrusen des Menschen und des Saugetieres, ihre Bedeutung sowie die Muscularis sexualis. Zoologica Stuttgart 72: 1–154.

[87] Hurley HJ, Shelley WB (1954) The role of myoepithelium of the human apocrine sweat gland. J Invest Derm 22: 143–155. 13130897 10.1038/jid.1954.19

[88] Rothman S (1954) Physiology and Biochemistry of the Skin. Chicago: University of Chicago Press. 741p.

[89] Kuno YA (1956) Human Perspiration. Illinois: Charles C Thomas Press. 416p.

[90] Montagna W (1956) The Structure and Function of the Skin. New York: Academic Press. 454p.

[91] Charles A (1959) An electron microscopic study of the human axillary apocrine gland.J Anat. 93: 226–232. PMC1244306 13641121

[92] Wilhelm B, Keppler C, Hoffbauer G, Lottspeich F, Linder D, et al (1998) Cytoplasmic carbonic anhydrase II of rat coagulating gland is secreted via the apocrine export mode. J Histochem Cytochem 46: 505–511. 9524196 10.1177/002215549804600410

[93] Stolle CA, McGowan MH, Heim RA, Varia M, Neubauer JA (1991) Nucleotide sequence of a cDNA encoding rat brain carbonic anhydrase II and its deduced amino acid sequence. Gene 109: 265–267. 1765271 10.1016/0378-1119(91)90619-m

[94] Seitz J, Keppler C, Rausch U, Aumüller G (1990) Immunohistochemistry of secretory transglutaminase from rodent prostate. Histochemistry 93: 525–530. 1970553 10.1007/BF00266412

[95] Steinhoff M, Eicheler W, Holterhus PM, Rausch U, Seitz J, et al (1994) Hormonally induced changes in apocrine secretion of transglutaminase in the rat dorsal prostate and coagulating gland. Eur J Cell Biol 65: 49–59. 7889995

[96] Manin M, Lecher P, Martinez A, Tournadre S, Jean C (1995) Exportation of mouse vas deferens protein, a protein without a signal peptide, from mouse vas deferens epithelium: a model of apocrine secretion. Biol Reprod 52: 50–62. 7711183 10.1095/biolreprod52.1.50

[97] Schaumburg-Lever G, Lever WF (1975) Secretion from human apocrine glands: an electron microscopic study. J Invest Dermatol 64: 38–41. 1110304 10.1111/1523-1747.ep12540893

[98] Main T, Lim D (1976) The human external auditory canal secretory system-an ultrastructural study. Laryngoscope 86: 1164–1176. 950858 10.1288/00005537-197608000-00008

[99] Stinson SF, Loosli CG (1978) Ultrastructural evidence concerning the mode of secretion of electron-dense granules by Clara cells. J Anat 127: 291–298. 721691 PMC1235769

[100] Smith JD, Hearn GW (1979) Ultrastructure of the apocrine sebaceous anal scent gland of the woodchuck, *Marmota monax*: evidence for apocrine and merocrine secretion by a single cell type. Anat Rec 193: 269–292. 426299 10.1002/ar.1091930208

[101] Satoh Y, Ishikawa K, Oomori Y, Takeda S, Ono K (1992) Secretion mode of theHarderian gland of rats after stimulation by cholinergic secretagogues. Acta Anat 143: 7–13. 1350161 10.1159/000147222

[102] Lucheroni A, Maurizi M, Spreca A, Palmerini CA, Binazzi M (1986) Some aspects of the secretory activity of the human olfactory glands. Rhinology 24: 57–60. 3704466

[103] Atoji Y, Yamamoto Y, Suzuki Y (1998) Apocrine sweat glands in the circumanal glands of the dog. Anat Rec 252: 403–412. 9811218 10.1002/(SICI)1097-0185(199811)252:3<403::AID-AR8>3.0.CO;2-F

[104] Aumüller G, Wilhelm B, Seitz J (1999) Apocrine secretion.fact or artifact? Anat Anz 181: 437–446. 10.1016/S0940-9602(99)80020-X 10560009

[105] Groos S, Wilhelm B, Renneberg H, Riva A, Reichelt R, et al (1999) Simultaneous apocrine and merocrine secretion in the rat coagulation gland. Cell Tissue Res 295: 495–504. 10022969 10.1007/s004410051255

[106] Baccari GC, Chieffi G, Di Mattea L, Dafnis D, De Rienzo G, et al (2000) Morphology of the Harderian gland of the Gecko (*Tarentola mauritanica*). J Morphol 244: 137–142. 10761051 10.1002/(SICI)1097-4687(200005)244:2<137::AID-JMOR4>3.0.CO;2-O

[107] Zaviačič M, Jakubovská V, Belošovič M, Breza J (2000) Ultrastructure of the normal adult human female prostate gland (Skene's glands). Anat Embryol 201: 51–61. 10603093 10.1007/pl00022920

[108] Payne AP (1994) The Harderian gland: a tercentenial review. J Anat 185: 1–49. 7559104 PMC1166813

[109] Gesase AP, Satoh Y, Ono K (1996) Secretagogue-induced apocrine secretion in the rat Harderian gland of the rat. Cell Tissue Res 285: 501–507. 8772164 10.1007/s004410050666

[110] Fraenkel G, Brookes VJ (1953) The process by which the puparia of many species of flies become fixed to a substrate. Biol Bull Mar Lab Woods Hole 105: 442–449.

[111] Lehmann M (1996) *Drosophila Sgs* genes: stage and tissue specificity of hormone responsiveness. BioEssays 18: 47–54. 8593163 10.1002/bies.950180110

[112] Korge G (1975) Chromosome puff activity and protein synthesis in larval salivary glandsof *Drosophila melanogaster* . Proc Natl Acad Sci USA 72: 4550–4554. 812097 10.1073/pnas.72.11.4550 PMC388760

[113] Korge G (1977) Larval saliva in *Drosophila melanogaster*: production, composition, and relationship to chromosome puffs. Dev Biol 58: 339–355. 407116 10.1016/0012-1606(77)90096-3

[114] Zhimulev IF, Kolesnikov NN (1975) Synthesis and secretion of mucoprotein glue in the salivary gland of *Drosophila melanogaster* . Wilhelm Roux's Archiv 178: 15–28. 10.1007/BF00848359 28305064

[115] Berendes HD, Ashburner M (1978) The salivary glands. In: Ashburner M, Wright TRF, editors. The Genetics and Biology of *Drosophila*, vol 2b. London and New York: Academic Press. pp. 453–498.

[116] Lane NJ, Carter YR, Ashburner M (1972) Puffs and salivary gland function: the fine structure of the larval and prepupal salivary glands of *Drosophila melanogaster* . Wilhelm Roux's Arch 169: 216–238. 10.1007/BF00582554 28304626

[117] Boyd M, Ashburner M (1977) The hormonal control of salivary gland secretion in *Drosophila melanogaster*: studies *in vitro* . J Insect Physiol 23: 517–523.

[118] Burtis KC, Thummel CS, Jones CW, Karim FD, Hogness DS (1990) The *Drosophila* 74EF early puff contains *E74*, a complex ecdysone-inducible gene that encodes two ets-related proteins. Cell 61: 85–99. 2107982 10.1016/0092-8674(90)90217-3

[119] Segraves WA, Hogness DS (1990) The *E75* ecdysone-inducible gene responsible for the 75B early puff in *Drosophila* encodes two new members of the steroid receptor superfamily. Genes Dev 4: 204–219. 2110921 10.1101/gad.4.2.204

[120] Thummel CS (1996) Flies on steroids - *Drosophila* metamorphosis and the mechanisms of steroid hormone action. Trends Genet 12: 306–310. 8783940 10.1016/0168-9525(96)10032-9

[121] Thummel CS (2002) Ecdysone-regulated puff genes 2000. Insect Biochem Mol Biol 32: 113–120. 11755052 10.1016/s0965-1748(01)00112-6

[122] von Gaudecker B (1972) Der Strukturwandel der larvalen Speicheldrüse von *Drosophila melanogaster*. Ein Beitrag zur Frage nach der steuernden Wirkung aktiver Gene auf das Cytoplasma. Z Zellforsch 127: 50–86. 4336139

[123] Sarmiento LA, Mitchell HK (1982) *Drosophila melanogaster* salivary gland proteins and pupation. Dev Genet 3: 255–272.

[124] Jiang C, Baehrecke EH, Thummel CS (1997) Steroid regulated programmed cell death during *Drosophila* metamorphosis. Development 124: 4673–4683. 9409683 10.1242/dev.124.22.4673

[125] Jiang C, Lamblin A-FJ, Steller H, Thummel CS (2000) A steroid-triggered transcriptional hierarchy controls salivary gland cell death during *Drosophila* metamorphosis. Molec Cell 5: 445–455. 10882130 10.1016/s1097-2765(00)80439-6

[126] Baehrecke EH (2003) Autophagic programmed cell death in *Drosophila* . Cell Death Differ 10: 940–945. 12934068 10.1038/sj.cdd.4401280

[127] Tissiéres A, Mitchell HK, Tracy UM (1974) Protein synthesis in salivary glands of *Drosophila melanogaster*: relation to chromosome puffs. J Mol Biol 84: 389–398. 4219221 10.1016/0022-2836(74)90447-1

[128] Zhimulev IF, Izquierdo ML, Lewis M, Ashburner M (1981) Patterns of protein synthesis in salivary glands of *Drosophila melanogaster* during larval and prepupal development. Roux's Arch Dev Biol 190: 351–357. 10.1007/BF00863272 28305294

[129] Ashburner M, Chihara C, Meltzer P, Richards G (1974) Temporal control of puffing activity in polytene chromosomes. Cold Spring Harbor Symp Quant Biol 38: 655–662. 4208797 10.1101/sqb.1974.038.01.070

[130] Richards GP (1976) Sequential gene activation by ecdysone in polytene chromosomes of *Drosophila melanogaster*. V. The late prepupal puffs. Dev Biol 54: 264–275. 825405 10.1016/0012-1606(76)90304-3

[131] Voges D, Zwickl P, Baumeister W (1999) The 26S proteasome: a molecular machine designed for controlled proteolysis. Annu Rev Biochem 68: 1015–1068. 10872471 10.1146/annurev.biochem.68.1.1015

[132] Davies KJ (2001) Degradation of oxidized proteins by the 20S proteasome. Biochimie 83: 301–310. 11295490 10.1016/s0300-9084(01)01250-0

[133] Orlowski M, Wilk S (2003) Ubiquitin-independent proteolytic functions of the proteasome. Arch Biochem Biophys 415: 1–5. 12801506 10.1016/s0003-9861(03)00197-8

[134] Fang S, Weissman AM (2004) A field guide to ubiquitylation. Cell Mol Life Sci 61: 1546–1561. 15224180 10.1007/s00018-004-4129-5 PMC11138666

[135] Husnjak K, Elsasser S, Zhang N, Chen X, Randles L, et al (2008) Proteasome subunit Rpn13 is a novel ubiquitin receptor. Nature 453: 481–488. 18497817 10.1038/nature06926 PMC2839886

[136] Su V, Lau AF (2009) Ubiquitin-like and ubiquitin-associated domain proteins: significance in proteasomal degradation. Cell Mol Life Sci 66: 2819–2833. 19468686 10.1007/s00018-009-0048-9 PMC2725189

[137] Maybeck V, Röper K (2009) A targeted gain-of-function screen identifies genes affecting salivary gland morphogenesis/tubulogenesis in *Drosophila* . Genetics 181: 543–565. 19064711 10.1534/genetics.108.094052 PMC2644946

[138] Chintapalli VR, Wang J, Dow JAT (2007) Using FlyAtlas to identify better *Drosophila melanogaster* models of human disease. Nat Genet 39: 715–720. 17534367 10.1038/ng2049

[139] Graveley BR, Brooks AN, Carlson JW, Duff MO, Landolin JM, et al (2011) The developmental transcriptome of *Drosophila melanogaster* . Nature 471: 473–479. 21179090 10.1038/nature09715 PMC3075879

[140] Ng V, Cho P, To C-H (2000) Tear proteins of normal young Hong Kong Chinese. Graefe‘s Arch Clin Exp Ophthalmol 238: 738–745. 11045341 10.1007/s004170000140

[141] Grus FH, Podust VN, Bruns K, Lackner K, Fu S, et al (2005) SELDI-TOF-MS ProteinChip array profiling of tears from patients with dry eye. Invest Ophthalmol Vis Sci 46: 863–876. 15728542 10.1167/iovs.04-0448

[142] Zhou L, Beuerman RW, Chan CM, Zhao SZ, Li XR, et al (2009) Identification of tear fluid biomarkers in dry eye syndrome using iTRAQ quantitative proteomics. J Proteome Res 8: 4889–4905. 19705875 10.1021/pr900686s

[143] Versura P, Nanni P, Bavelloni A, Blalock WL, Piazzi M, et al (2010) Tear proteomics in evaporative dry eye disease. Eye 24: 1396–1402. 20150925 10.1038/eye.2010.7

[144] Wrobel A, Seltamnn H, Fimmel S, Müller-Decker K, Tsukada M, et al (2003) Differentiation and apoptosis in human immortalized sebocytes. J Invest Dermatol 120: 175–181. 12542519 10.1046/j.1523-1747.2003.12029.x

[145] Horsley W, O'Carroll D, Tooze R, Ohinata Y, Saitou M, et al (2006) Blimp1 defines a progenitor population that governs cellular input to the sebaceous gland. Cell 126: 597–609. 16901790 10.1016/j.cell.2006.06.048 PMC2424190

[146] Schneider MR, Paus R (2010) Sebocytes, multifaceted epithelial cells: Lipid production and holocrine secretion. Intl J Bioch Cell Biol 42: 181–185. 10.1016/j.biocel.2009.11.017 19944183

